# Investigation into the efficacy and safety profile of oral small-molecule GLP-1 receptor agonists in type 2 diabetes and obesity: a systematic review and meta-analysis

**DOI:** 10.3389/fendo.2026.1854779

**Published:** 2026-07-07

**Authors:** Li Li, Dianpeng Shui, Xiong Zhang, Bing Tan, Yan Deng

**Affiliations:** 1 Hainan Vocational University of Science and Technology, Haikou, China; 2 Department of Pharmacy, Affiliated Hospital of Youjiang Medical University for Nationalities, Baise, Guangxi, China; 3 National Immunological Laboratory of Traditional Chinese Medicine, Affiliated Hospital of Youjiang Medical University for Nationalities, Baise, Guangxi, China; 4 Department of Obstetrics and Gynecology, Affiliated Hospital of Youjiang Medical University for Nationalities, Baise, Guangxi, China

**Keywords:** danuglipron, meta-analysis, obesity, oral small-molecule GLP-1RAs, orforglipron, type 2 diabetes

## Abstract

**Background:**

This meta-analysis aimed to evaluate the efficacy and safety of oral small-molecule glucagon-like peptide-1 receptor agonists (GLP-1RAs) in type 2 diabetes (T2D) and obesity.

**Methods:**

We performed a meta-analysis of randomized controlled trials assessing the efficacy and safety of oral small-molecule GLP-1RAs in adults with T2D or obesity. A systematic literature search was conducted in databases including Web of Science, PubMed, Scopus, Google Scholar, Embase, and the Cochrane Library from database inception to March 2026. Continuous outcomes were summarized as mean differences (MDs), and dichotomous outcomes as risk ratios (RRs).

**Results:**

Ten studies were included. Oral small-molecule GLP-1RAs significantly reduced body weight (MD=-3.93, 95%CI:-4.78 to -3.09), body mass index (MD=-2.39, 95%CI:-3.02 to -1.77), waist circumference (MD=-4.62, 95%CI:-6.09 to -3.16), fasting blood glucose (MD=-24.59, 95%CI:-28.80 to -20.37), and HbA1c (MD=-0.94, 95%CI: -1.09 to -0.79), while fasting insulin was not significantly changed (MD = 1.21, 95%CI: -0.77 to 3.19). These agents increased likelihood of achieving weight loss, including ≥5% (RR = 2.68, 95% CI: 2.24 to 3.20), ≥10% (RR = 4.14, 95% CI: 3.19 to 5.36), and ≥15% body weight reduction (RR = 10.61, 95% CI: 7.76 to 14.49). Treatment-emergent adverse events (RR = 1.09, 95% CI: 1.06 to 1.12) and overall adverse events (AEs) (RR = 2.75, 95% CI: 2.41 to 3.15) were increased, particularly gastrointestinal events such as nausea, vomiting, diarrhea, constipation, dyspepsia, and abdominal symptoms. However, serious adverse events were not increased (RR = 1.15, 95%CI: 0.93 to 1.43).

**Conclusions:**

Oral small-molecule GLP-1RAs provide significant benefits in weight reduction and glycemic control, supporting their potential role as effective oral incretin therapies. However, their clinical use, should balance these metabolic benefits against high incidence of predominantly gastrointestinal AEs.

## Introduction

Glucagon-like peptide-1 receptor agonists (GLP-1RAs) have become an important therapeutic class for managing type 2 diabetes (T2D) and obesity because they improve glycemic control, promote weight reduction, and provide broader cardiometabolic benefits (1). Conventional GLP-1RAs were initially developed as peptide-based injectable agents. Although oral semaglutide provides a non-injectable option, it remains a peptide formulation that requires an absorption enhancer and strict administration conditions, including fasting and water restrictions (2, 3). These limitations have driven oral small-molecule GLP-1RAs development, which may preserve the metabolic benefits of GLP-1 receptor activation while offering simpler administration and potentially improved acceptability and accessibility (4–6).

Among these emerging agents, danuglipron and orforglipron are the most extensively studied oral small-molecule GLP-1RAs. Early clinical studies of danuglipron demonstrated reductions in fasting plasma glucose, glycated hemoglobin (HbA1c), and body weight in adults with T2D, with adverse events (AEs) consistent with known GLP-1RA class profile (7–10). Similarly, phase 1 and phase 2 studies of orforglipron showed reductions in HbA1c and body weight, supporting its further development as oral non-peptide GLP-1RA (11–13). More recent longer-term studies have suggested that orforglipron may achieve clinically relevant reductions in body weight and glycemic indices in patients with obesity and/or T2D, potentially approaching the efficacy seen with established injectable incretin therapies (14–16).

Available randomized trials indicate that oral small-molecule GLP-1RAs can improve clinically relevant outcomes. In addition to improving glycemic control, weight reduction of 5%, 10%, or greater are clinically meaningful because greater weight loss is associated with progressive improvements in metabolic comorbidities. Prior studies of injectable and oral incretin-based therapies, including liraglutide, semaglutide, and tirzepatide, have established the central role of this therapeutic class in achieving meaningful weight reduction and metabolic improvement, thereby providing an important clinical context for evaluating danuglipron and orforglipron (15, 16).

Safety and tolerability remain equally important considerations for this emerging drug class. Across published trials, the most frequently reported adverse events (AEs) with oral small-molecule GLP-1RAs have been gastrointestinal, particularly nausea, vomiting, diarrhea, constipation, dyspepsia, and related abdominal symptoms, with many events occurring during dose escalation. Although most events have been mild to moderate and consistent with the established GLP-1RA class profile, these AEs may influence treatment adherence, dose optimization, and long-term persistence (16–18). Therefore, a meta-analysis is needed to comprehensively assess the efficacy and safety of oral small-molecule GLP-1RAs and to clarify overall benefit-risk profile of danuglipron and orforglipron across randomized controlled trials (RCTs).

## Methods

### Search strategy

**Figure 1:**
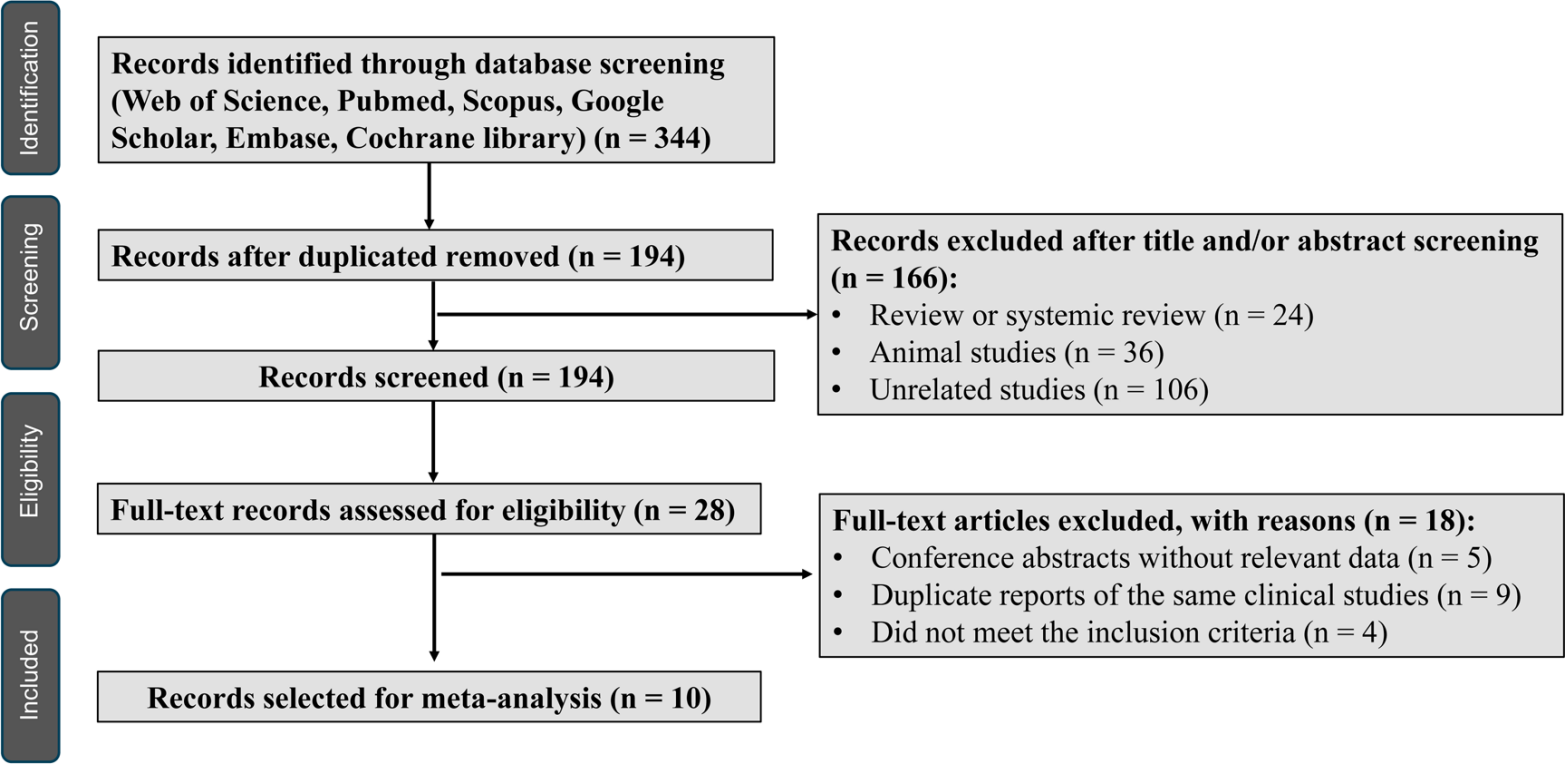
Flow diagram of study selection.

A systematic literature search was conducted to identify RCTs evaluating the efficacy and safety of oral small-molecule GLP-1RAs in adults with type 2 diabetes (T2D), T2D with obesity, or overweight/obesity without diabetes. A systematic literature search was conducted in databases including Web of Science, PubMed, Scopus, Google Scholar, Embase, and the Cochrane Library from database inception to March 2026. The search strategy combined controlled vocabulary and free-text terms related to the intervention and target conditions. The main search terms included: “danuglipron”, “orforglipron”, “oral small-molecule GLP-1 receptor agonist”, “GLP-1 receptor agonist”, “glucagon-like peptide-1 receptor agonist”, “type 2 diabetes”, “obesity”, “overweight”, “glycemic control”, “body weight”, and “randomized controlled trial”. Boolean operators, including AND and OR, were used to combine terms appropriately. After records retrieval, duplicate publications were removed, followed by screening of titles and abstracts. Potentially eligible studies then underwent full-text assessment. The study selection process was presented using a PRISMA flow diagram (**Figure 1**).

### Eligibility criteria

Studies were included if they met the following criteria: RCT design; enrolment of adult participants with T2D, T2D with obesity, or overweight/obesity without diabetes; evaluation of oral small-molecule GLP-1RAs, including danuglipron or orforglipron; and reporting of extractable data for at least one efficacy or safety outcome. Studies were excluded if they were conference abstracts without sufficient quantitative data, animal studies, reviews or systematic reviews, duplicate reports of the same trial, or otherwise did not meet the inclusion criteria.

### Outcomes of interest

The efficacy outcomes of interest included body weight change, achievement of ≥5%, ≥10%, and ≥15% body weight reduction, body mass index (BMI), waist circumference, fasting blood glucose (FBG), fasting insulin (FINs), and HbA1c. Safety outcomes included treatment-emergent AEs (TEAEs), AEs, serious AEs (SAEs), and specific gastrointestinal adverse events, including nausea, vomiting, diarrhea, constipation, dyspepsia, decreased appetite, eructation, gastro-esophageal reflux disease, abdominal distension, abdominal discomfort, and abdominal pain.

### Data extraction

Data were independently extracted from the eligible studies and summarized in **Table 1**. Extracted information included study name, trial phase, study population, intervention, comparator, treatment duration, sample size, and baseline participant characteristics. Baseline characteristics included age, body weight, BMI, HbA1c, and fasting plasma glucose, where available. When multiple reports described the same clinical trial, the most appropriate and non-overlapping dataset was selected for quantitative synthesis.

**Table 1:** Study characteristics of included studies.

Study year	Study design	Patients	Intervention	Sample size (female/male)	Mean age (years old)	Body weight (kg)	BMI (kg/m2)	Baseline HbA1c (%)	Baseline FPG (mg/dl)	Disease duration (years)	Treatment duration
Frias 2024 (13)	RCT, multicentre, double-blind, Phase II	Type 2 diabetes	Placebo	55 (27/28)	58.3 ± 9.5	102.0 ± 18.8	35.8 ± 6.2	8.1 ± 0.9	172.0 ± 42.9	7.8	26 weeks
			Orforglipron (3 mg, qd)	51 (25/26)	59.0 ± 9.4	99.3 ± 25.4	35.3 ± 8.2	8.0 ± 0.8	164.0 ± 40.9	5	
			Orforglipron (12 mg, qd)	56 (20/36)	57.4 ± 9.2	99.3 ± 18.1	34.8 ± 6.3	8.2 ± 0.9	172.1 ± 42.8	7.1	
			Orforglipron (24 mg, qd)	47 (17/30)	60.5 ± 9.1	98.5 ± 22.9	34.1 ± 7.7	8.2 ± 0.9	171.7 ± 44.4	5.9	
			Orforglipron (36 mg, qd)	61 (25/36)	59.7 ± 9.2	98.9 ± 17.5	34.4 ± 5.4	8.0 ± 0.7	157.9 ± 28.7	5.9	
			Orforglipron (45 mg, qd)	63 (23/40)	58.5 ± 9.4	104.6 ± 25.1	36.4 ± 6.9	8.1 ± 0.9	166.4 ± 35.0	6.8	
Horn 2025 (16)	RCT, multicentre, double-blind, Phase III	Type 2 diabetes with obesity	Placebo	630 (298//332)	56.5 ± 10.9	101.2 ± 22.6	35.5 ± 6.5	8.03 ± 0.75	151.5 ± 39.5	6.8	72 weeks
			Orforglipron (6 mg, qd)	329 (150/179)	56.8 ± 10.4	102.3 ± 22.7	35.9 ± 7.0	8.03 ± 0.73	152.9 ± 41.6	7.6	
			Orforglipron (12 mg, qd)	332 (155/177)	56.2 ± 10.5	102.7 ± 21.3	36.1 ± 6.3	8.08 ± 0.76	155.1 ± 43.0	6.4	
			Orforglipron (36 mg, qd)	322 (154/168)	58.1 ± 10.8	99.8 ± 23.0	35.1 ± 6.5	8.05 ± 0.73	154.7 ± 40.1	7	
Ohwaki 2026 (19)	RCT, double-blind, placebo-controlled, Phase I	Type 2 diabetes	Placebo	11 (1/10)	57.7 ± 5.8	79.4 ± 10.1	27.6 ± 3.7	7.8 ± 1.1	184.0 ± 55.3	9.4	12 weeks
			Orforglipron (12 mg, daily)	14 (1/13)	56.3 ± 8.6	82.8 ± 9.5	28.3 ± 3.3	8.0 ± 0.8	175.9 ± 33.7	6.1	
			Orforglipron (24 mg, daily)	17 (0/17)	53.4 ± 10.0	80.8 ± 15.2	27.2 ± 4.5	7.9 ± 0.7	173.8 ± 29.8	5.5	
			Orforglipron (45 mg, daily)	18 (0/18)	57.6 ± 6.2	74.7 ± 16.3	26.1 ± 4.7	7.8 ± 0.9	188.0 ± 47.3	8.9	
Ono 2023 (10)	RCT, double-blind, placebo-controlled, Phase I	Type 2 diabetes	Placebo	9 (1/8)	58.6 ± 8.8	73.3 ± 9.9	25.9 ± 2.7	8.3 ± 1.2	182.9 ± 36.3	5.5	8 weeks
			Danuglipron (40 mg, BID)	10 (2/8)	55.9 ± 10.0	79.7 ± 13.6	28.6 ± 4.1	8.2 ± 1.1	173.3 ± 29.7	2.9	
			Danuglipron (80, mg, BID)	9 (1/8)	58.0 ± 6.7	79.6 ± 10.0	28.2 ± 3.6	8.6 ± 1.0	175.7 ± 32.7	9.1	
			Danuglipron (120 mg, BID)	9 (1/8)	50.7 ± 7.5	81.5 ± 10.4	28.6 ± 3.4	8.4 ± 1.2	163.7 ± 47.5	5.7	
Pratt 2023 (11)	RCT, multicentre, double-blind, placebo-controlled, Phase 1b	Type 2 diabetes	Placebo	17 (10/7)	56.0 ± 6.0	90.29 ± 20.04	31.31 ± 4.86	8.09 ± 0.75		8.63	12 weeks
			Orforglipron (9 mg, daily)	9 (5/4)	57.7 ± 6.4	85.61 ± 12.76	30.14 ± 3.60	8.02 ± 0.62		13.48	
			Orforglipron (15 mg, daily)	10 (3/7)	59.6 ± 4.6	88.02 ± 14.36	30.39 ± 3.61	7.84 ± 0.74		15.02	
			Orforglipron (21 mg, daily)	14 (4/10)	55.3 ± 8.0	92.09 ± 18.78	32.60 ± 5.48	8.36 ± 1.31		9.48	
			Orforglipron (27 mg, daily)	9 (2/7)	58.8 ± 4.6	92.80 ± 15.36	30.62 ± 3.55	7.82 ± 0.69		7.6	
			Orforglipron (45 mg, daily)	9 (5/4)	62.8 ± 4.4	81.49 ± 10.24	29.82 ± 2.84	7.93 ± 0.79		10.38	
Rosenstock 2025 (20)	RCT, double-blind, placebo-controlled, Phase III	Type 2 diabetes	Placebo	138 (63/75)	53.3 ± 12.5	90.0 ± 20.7	32.9 ± 6.8	7.96 ± 0.89	143.3 ± 42.2	4.4	40 weeks
			Orforglipron (3 mg, daily)	143 (63/80)	53.3 ± 11.3	90.3 ± 25.7	32.9 ± 8.0	7.93 ± 0.86	142.9 ± 38.7	4	
			Orforglipron (12 mg, daily)	137 (71/66)	54.1 ± 11.8	90.6 ± 23.1	33.3 ± 7.8	7.98 ± 0.91	155.3 ± 55.1	5.1	
			Orforglipron (36 mg, daily)	141 (72/69)	52.8 ± 11.8	90.1 ± 22.9	33.1 ± 7.3	8.07 ± 0.90	148.8 ± 40.0	4.2	
Saxena 2021 (7)	RCT, double-blind, placebo-controlled, Phase I	Type 2 diabetes	Placebo	25 (13/12)	57.6 ± 7.7	94.3 ± 17.7	33.2 ± 4.5	8.0 ± 0.80	167.6 ± 32.5	8.5	4 weeks
			Danuglipron 10 mg BID	9 (2/7)	54.0 ± 6.2	99.6 ± 12.7	33.9 ± 3.4	8.2 ± 0.6	158.3 ± 23.3	11.7	
			Danuglipron 15 mg BID	9 (6/3)	56.1 ± 9.5	92.9 ± 22.5	35.3 ± 5.1	8.6 ± 0.6	198.4 ± 33.1	8.1	
			Danuglipron 50 mg BID	10 (5/5)	60.2 ± 8.0	87.8 ± 13.8	32.6 ± 3.6	8.07 ± 0.9	168.1 ± 34.9	9.1	
			Danuglipron 70 mg BID	9 (5/4)	58.3 ± 5.3	86.9 ± 18.6	31.9 ± 4.3	8.3 ± 0.6	186.3 ± 32.0	11.6	
			Danuglipron (120 mg, BID)-1	9 (2/7)	55.8 ± 7.7	101.6 ± 17.4	35.0 ± 4.7	8.5 ± 1.0	196.9 ± 26.1	10.8	
			Danuglipron (120 mg, BID)-2	9 (5/4)	58.6 ± 6.7	87.9 ± 19.7	30.7 ± 4.5	8.2 ± 0.7	176.3 ± 44.0	9.2	
			Danuglipron (120 mg, BID)-3	8 (4/4)	58.3 ± 3.8	84.9 ± 15.3	31.3 ± 3.3	8.2 ± 1.0	179.9 ± 28.5	9.2	
			Danuglipron (200 mg BID)	10 (5/5)	57.6 ± 8.3	92.4 ± 18.0	31.9 ± 3.6	8.6 ± 1.0	195.3 ± 37.5	8.7	
Saxena 2023-a (9)	RCT, double-blind, placebo-controlled, Phase II	Type 2 diabetes	Placebo	16 (8/8)	53.9 ± 9.10	101.017 ± 18.44	35.5 ± 5.93	7.83 ± 0.936	162.6 ± 44.68	8.15	16 weeks
			Danuglipron (80 mg, BID)-1	20 (10/10)	59.5 ± 9.55	96.410 ± 20.43	35.1 ± 5.96	8.14 ± 1.025	178.3 ± 38.01	9.33	
			Danuglipron (80 mg, BID)-2	22 (10/12)	60.9 ± 8.69	91.111 ± 14.22	32.8 ± 5.51	8.25 ± 1.019	177.5 ± 44.34	10.38	
			Danuglipron (120 mg, BID)-1	22 (10/12)	58.3 ± 7.11	101.880 ± 24.54	35.1 ± 6.89	8.05 ± 0.880	159.8 ± 48.52	9.15	
			Danuglipron (120 mg, BID)-2	22 (10/12)	57.2 ± 11.80	95.152 ± 17.01	33.9 ± 4.47	8.56 ± 1.162	182.9 ± 57.50	10.2	
			Danuglipron (200 mg, BID)-1	21 (10/11)	59.0 ± 9.31	86.365 ± 13.64	31.5 ± 3.76	8.24 ± 1.162	167.7 ± 53.90	7.87	
		Obesity without diabetes	Placebo	6 (4/2)	49.5 ± 5.79	103.775 ± 15.76	36.7 ± 1.55	5.77 ± 0.258	95.8 ± 8.22	NA	
			Danuglipron (200 mg, BID)-2	22 (16/6)	48.5 ± 13.12	103.439 ± 12.72	37.4 ± 4.55	5.41 ± 0.413	104.5 ± 11.40	NA	
Saxena 2023-b (8)	RCT, double-blind, placebo-controlled, Phase IIb	Type 2 diabetes	Placebo	66 (33/33)	57.9 ± 10.27	90.1 ± 17.54	32.5 ± 5.08	8.24 ± 0.90	173.0 ± 43.74	8.8	16 weeks
			Danuglipron (2.5 mg, BID)	68 (30/38)	58.9 ± 9.30	90.9 ± 20.13	32.5 ± 5.17	8.10 ± 1.03	169.3 ± 42.40	8.8	
			Danuglipron (10 mg, BID)	68 (33/35)	58.1 ± 9.43	92.3 ± 16.44	33.0 ± 5.34	8.01 ± 0.91	165.4 ± 39.08	8.5	
			Danuglipron (40 mg, BID)	71 (37/34)	59.6 ± 8.58	90.2 ± 18.74	32.3 ± 5.25	8.00 ± 0.89	166.0 ± 39.33	8	
			Danuglipron (80 mg, BID)	67 (32/35)	58.4 ± 9.18	91.3 ± 16.64	32.9 ± 5.06	8.07 ± 0.95	172.8 ± 45.47	9.7	
			Danuglipron (120 mg, BID)	71 (37/34)	58.8 ± 9.43	93.1 ± 17.95	33.3 ± 5.70	8.05 ± 0.86	169.5 ± 40.65	8.7	
Wharton 2023 (14)	RCT, double-blind, placebo-controlled, Phase II	Obesity or overweight without diabetes	Placebo	58 (29/29)	49.8 ± 10.5	107.5 ± 25.3	37.7 ± 7.7	5.5 ± 0.4	94.4 ± 9.8	NA	36 weeks
			Orforglipron (12 mg, daily)	50 (31/19)	57.0 ± 9.1	112.1 ± 30.2	38.1 ± 7.7	5.7 ± 0.3	97.5 ± 12.0	NA	
			Orforglipron (24 mg, daily)	57 (30/27)	56.3 ± 11.8	107.8 ± 22.5	38.0 ± 6.4	5.7 ± 0.4	95.7 ± 13.2	NA	
			Orforglipron (36 mg, daily)-1	62 (18/44)	55.4 ± 10.9	108.8 ± 28.5	38.0 ± 6.3	5.6 ± 0.4	98.0 ± 13.5	NA	
			Orforglipron (36 mg, daily)-2	62 (18/44)	56.5 ± 10.7	105.2 ± 20.4	36.8 ± 5.5	5.7 ± 0.3	98.0 ± 8.5	NA	
			Orforglipron (45 mg, daily)-1	61 (19/42)	50.9 ± 12.6	110.9 ± 28.1	38.7 ± 7.6	5.6 ± 0.4	92.3 ± 10.1	NA	
			Orforglipron (45 mg, daily)-2	53 (16/37)	54.0 ± 8.8	107.6 ± 25.2	37.8 ± 6.5	5.6 ± 0.4	97.2 ± 10.2	NA	

### Risk of bias assessment

The methodological quality of the eligible studies was appraised using the Cochrane Risk of Bias tool, which evaluates key domains including detection bias, reporting bias, selection bias, attrition bias, performance bias, and other possible sources of bias. Each study was judged as having a low, high, or unclear risk of bias.

### Statistical analysis

Meta-analysis was performed for both efficacy and safety outcomes. For continuous variables, pooled effect sizes were calculated as mean differences (MDs) with 95% confidence intervals (CIs). For dichotomous outcomes, pooled estimates were expressed as risk ratios (RRs) with 95% CIs. Statistical heterogeneity among studies was assessed using the Cochran Q test and quantified with the I² statistic. A random-effects model was employed when heterogeneity was present, whereas a fixed-effects model was employed when heterogeneity was low. The choice of effect model was based on the degree of between-study heterogeneity. Subgroup analyses were performed according to danuglipron and orforglipron, where sufficient data were available. These analyses explored potential sources of heterogeneity and to compare the efficacy and safety profiles of two compounds across outcomes. Meta-regression analysis of body weight change and FBG according to drug doses were performed using Python 3.11.

### Publication bias

Publication bias was evaluated through funnel plots. Funnel plots were constructed for the main efficacy outcomes and for the analyzed adverse-event outcomes, as presented in the supplementary figures.

## Results

### Study selection

The literature search identified 344 records. After duplicate removal, 194 records remained for screening. After abstract and title screening, 166 records were excluded, including 24 reviews or systematic reviews, 36 animal studies, and 106 unrelated studies. Subsequently, 28 full-text articles were evaluated for eligibility, of which 18 were excluded, including five conference abstracts without relevant data, nine duplicate reports of the same clinical studies, and four studies that did not meet the inclusion criteria. Finally, 10 studies were included in the meta-analysis (**Figure 1**).

### Study characteristics

The study characteristics are presented in **Table 1**. Overall, 10 RCTs were included, comprising Phase I to Phase III studies evaluating danuglipron or orforglipron in adults with T2D, T2D with obesity, or obesity/overweight without diabetes. Treatment duration ranged from 4 to 72 weeks, and sample sizes varied from small early-phase cohorts to large multicenter phase III trials. Across studies, participants were generally middle-aged and had elevated baseline body weight and BMI, consistent with overweight or obesity. In trials involving participants with T2D, baseline HbA1c and FBG were above normal ranges, indicating inadequate glycemic control at study entry. Differences in study phase, patient population, treatment duration, and dose escalation schemes may have contributed to between-study heterogeneity.

### Risk of bias

**Figure 2:**
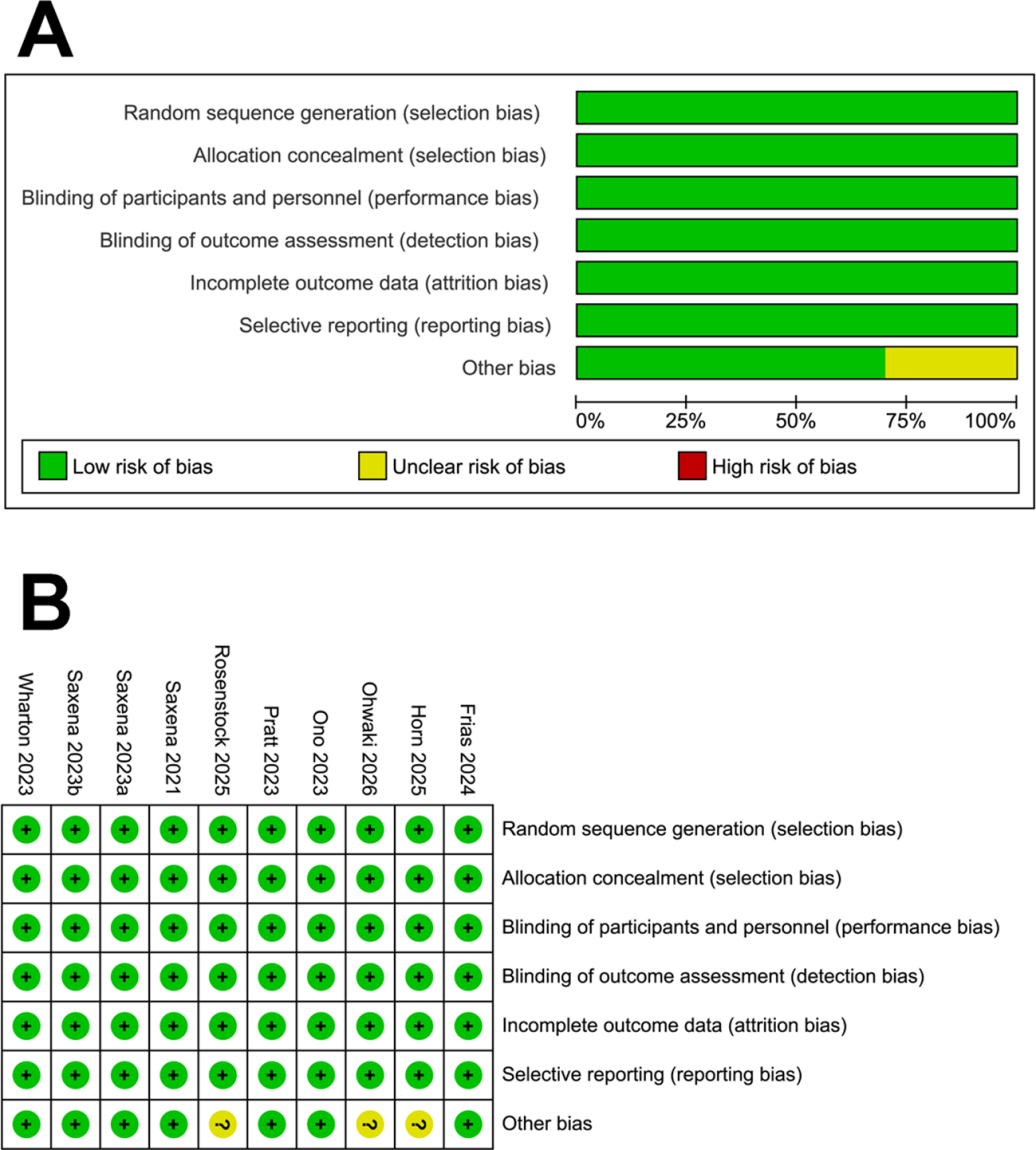
Risk-of-bias assessment of included studies. **(A)** Risk of bias graph. **(B)** Risk of bias summary.

The quality of included studies is presented in **Figure 2**. Overall, the risk of bias across studies was low. Overall, the included studies were judged to have a generally low risk of bias across the main domains, including selection, performance, detection, attrition, and reporting bias. However, in the ‘other bias’ domain, Horn 2025, Ohwaki 2026, and Rosenstock 2025 were classified as unclear risk. Therefore, the overall methodological quality was considered acceptable for quantitative synthesis, although minor uncertainties remained in selected domains.

### Body weight change

**Figure 3:**
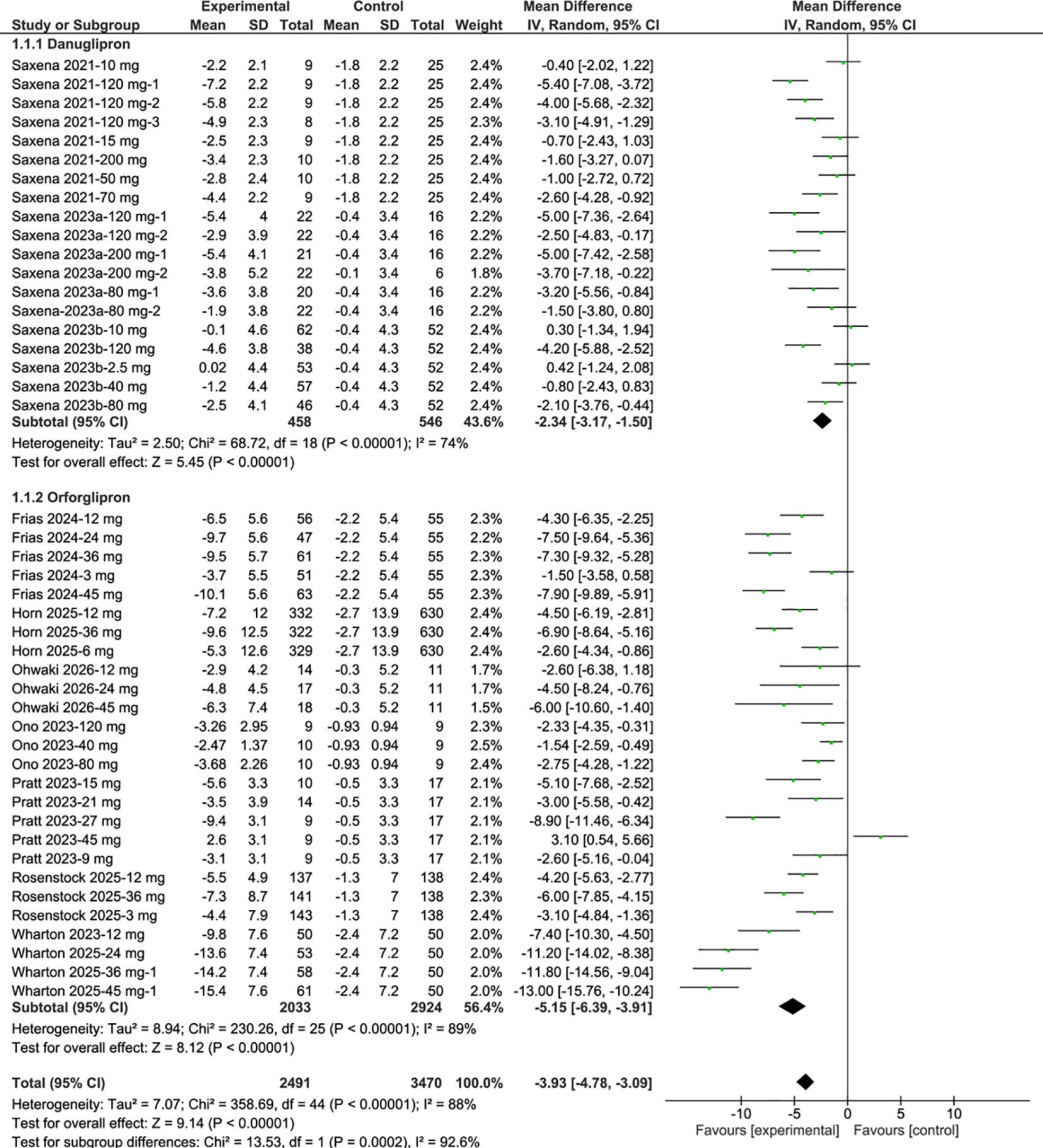
Forest plot of body weight change. Forest plot showing the pooled effect of oral small-molecule GLP-1 receptor agonists on body weight compared with control. Subgroup analyses were performed according to study drug (danuglipron and orforglipron).

Compared with control treatment, oral small-molecule GLP-1RAs significantly reduced body weight (MD =-3.93, 95%CI:-4.78 to -3.09; P<0.00001; **Figure 3**), although heterogeneity was observed in the overall analysis (I²=88%, heterogeneity P<0.00001). In subgroup analyses, both danuglipron and orforglipron were associated with body weight reduction. Danuglipron reduced body weight by -2.34 kg (95%CI:-3.17 to -1.50; P<0.00001), with heterogeneity (I² =74%, heterogeneity P<0.00001), whereas orforglipron showed a larger pooled effect (MD=-5.15, 95%CI: 6.39 to -3.91; P<0.00001), also with heterogeneity (I²=89%, heterogeneity P<0.00001).

### Categorical body weight reduction

**Figure 4:**
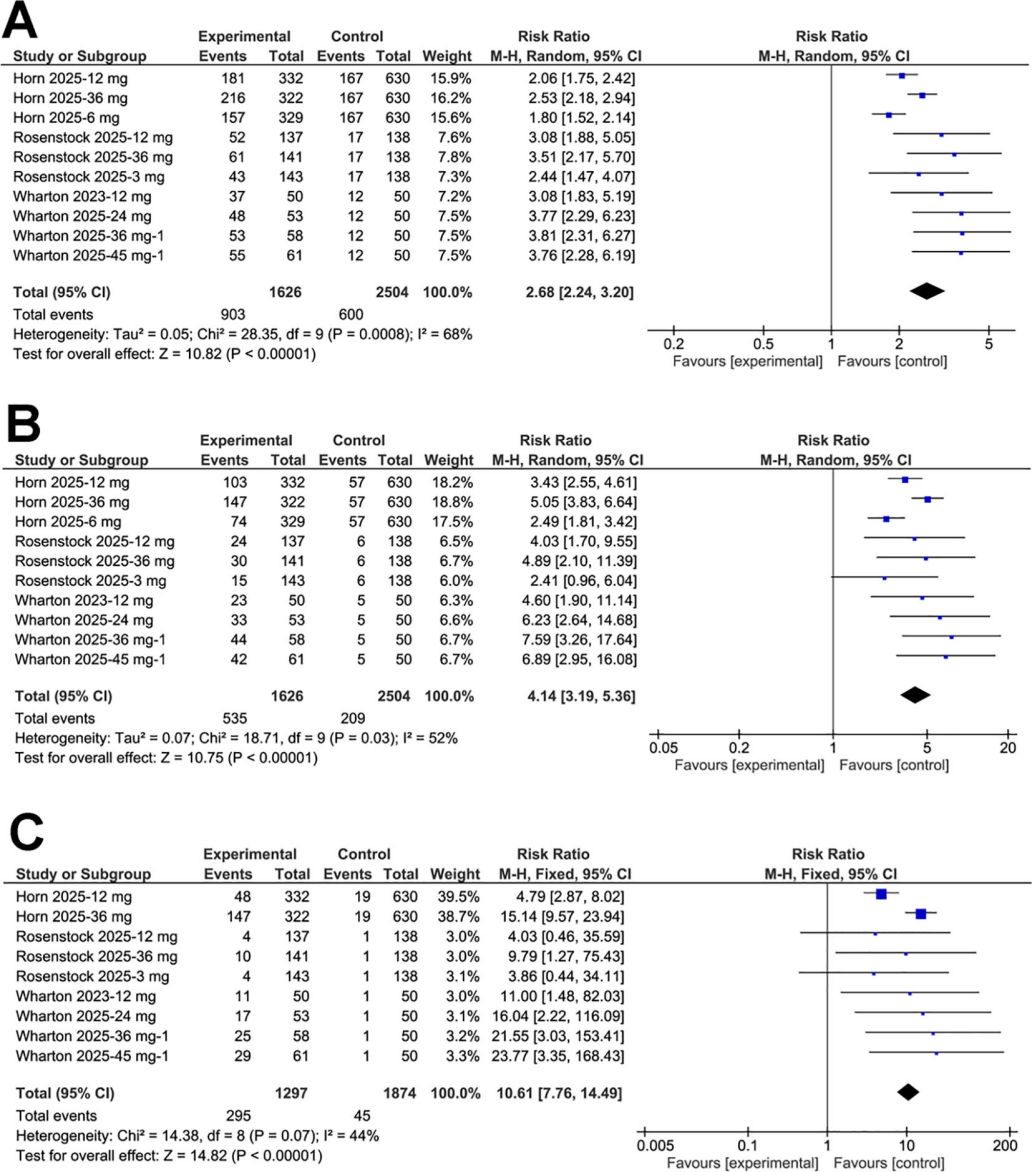
Forest plots of categorical body weight reduction. **(A)** Proportion of participants achieving ≥5% body weight reduction. **(B)** Proportion of participants achieving ≥10% body weight reduction. **(C)** Proportion of participants achieving ≥15% body weight reduction. Subgroup analyses were performed according to study drug (danuglipron and orforglipron).

Oral small-molecule GLP-1RAs increased the likelihood of weight-loss targets (**Figures 4A–C**). For ≥5% body weight reduction, the pooled RR was 2.68 (95%CI: 2.24 to 3.20; P<0.00001), with heterogeneity (I²=68%, heterogeneity P = 0.0008). For ≥10% body weight reduction, pooled RR was 4.14 (95%CI: 3.19 to 5.36; P<0.00001), with heterogeneity (I²=52%, heterogeneity P = 0.03). For ≥15% body weight reduction, the pooled RR was 10.61 (95%CI: 7.76 to 14.49; P<0.00001), with low heterogeneity (I²=44%, heterogeneity P = 0.07). Overall, these findings indicate that oral small-molecule GLP-1RAs improved the likelihood of achieving weight loss, with larger effect estimates at higher weight-loss thresholds.

### BMI and waist circumference

**Figure 5:**
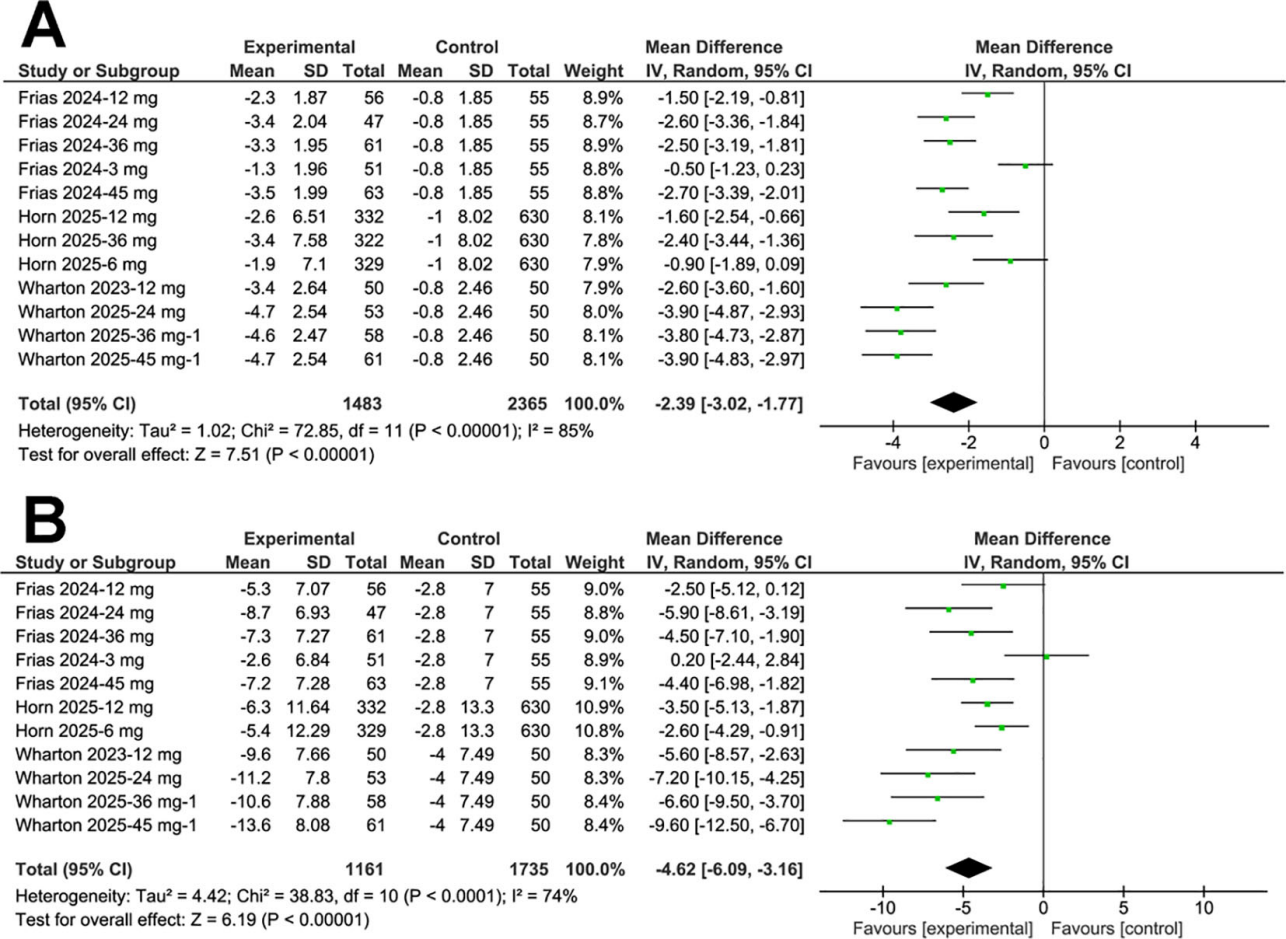
Forest plots of anthropometric outcomes. **(A)** Body mass index (BMI). **(B)** Waist circumference. Subgroup analyses were performed according to study drug (danuglipron and orforglipron).

Consistent with the body weight findings, oral small-molecule GLP-1RAs significantly reduced both BMI and waist circumference compared with control treatment (**Figures 5A, B**). The pooled MD for BMI was -2.39 (95 CI:-3.02 to -1.77; P<0.00001), with substantial heterogeneity (I²=85%, heterogeneity P<0.00001). Waist circumference was also reduced (MD=-4.62, 95%CI:-6.09 to -3.16; P < 0.00001), again with heterogeneity (I²=74%, heterogeneity P<0.0001).

### FBG and FINS

**Figure 6:**
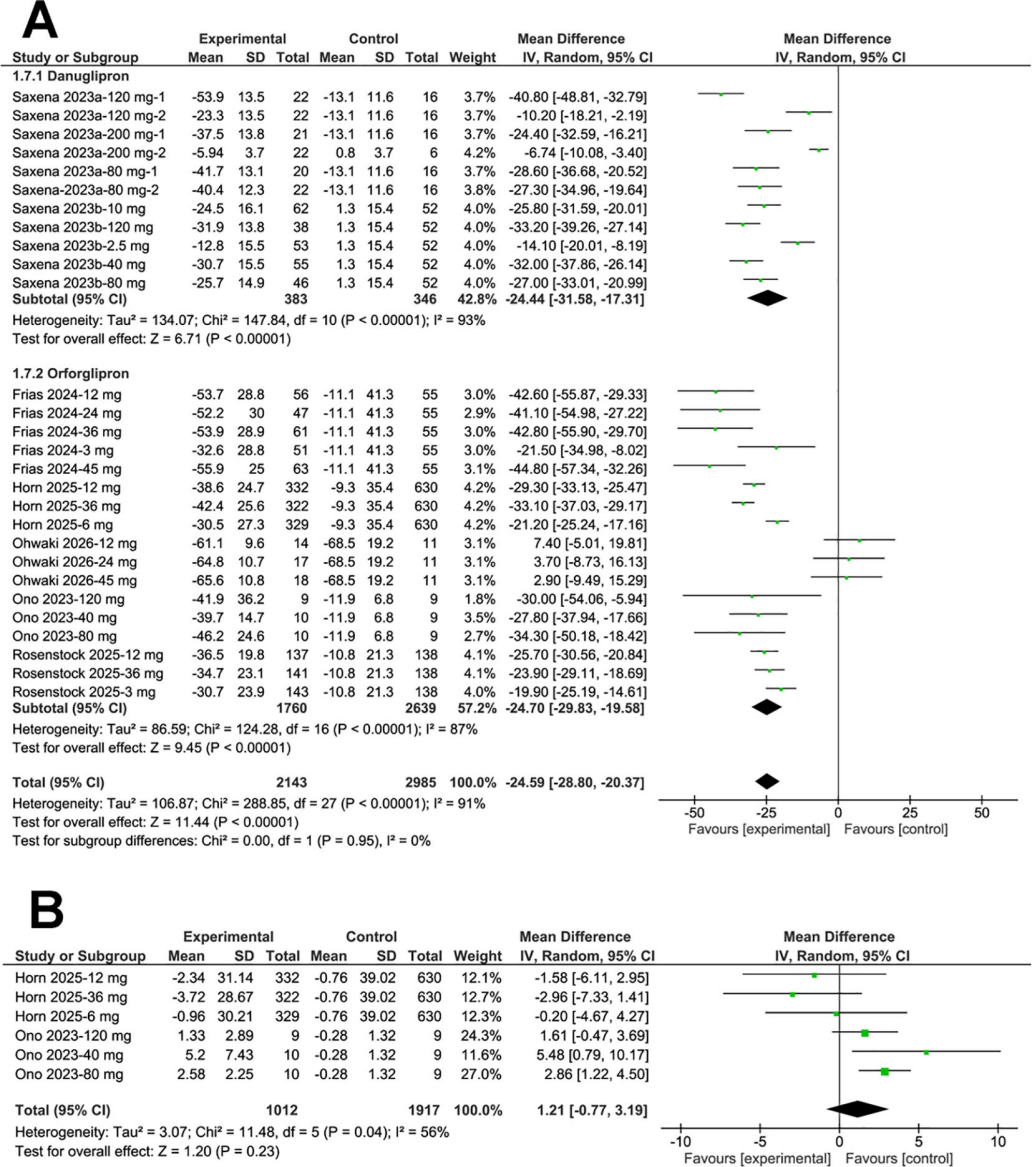
Forest plots of glycemic parameters. **(A)** Fasting blood glucose (FBG). **(B)** Fasting insulin (FINS). Subgroup analyses were performed according to study drug (danuglipron and orforglipron).

For glycemic outcomes, oral small-molecule GLP-1RAs significantly reduced FBG, but did not significantly affect FINS (**Figures 6A, B**). The pooled MD for FBG was -24.59 (95%CI: -28.80 to -20.37; P<0.00001), with marked heterogeneity (I²=91%, heterogeneity P<0.00001). In subgroup analyses, both danuglipron (MD=-24.44, 95%CI: -31.58 to -17.31; P<0.00001; I² = 93%, heterogeneity P<0.00001) and orforglipron (MD=-24.70, 95%CI: -29.83 to -19.58; P<0.00001; I²=87%, heterogeneity P<0.00001) reduced FBG, with broadly comparable effect sizes. By contrast, FINS was not significantly altered relative to control treatment (MD = 1.21, 95%CI: -0.77 to 3.19; P = 0.23), despite moderate heterogeneity (I²=56%, heterogeneity P = 0.04).

### HbA1c

**Figure 7:**
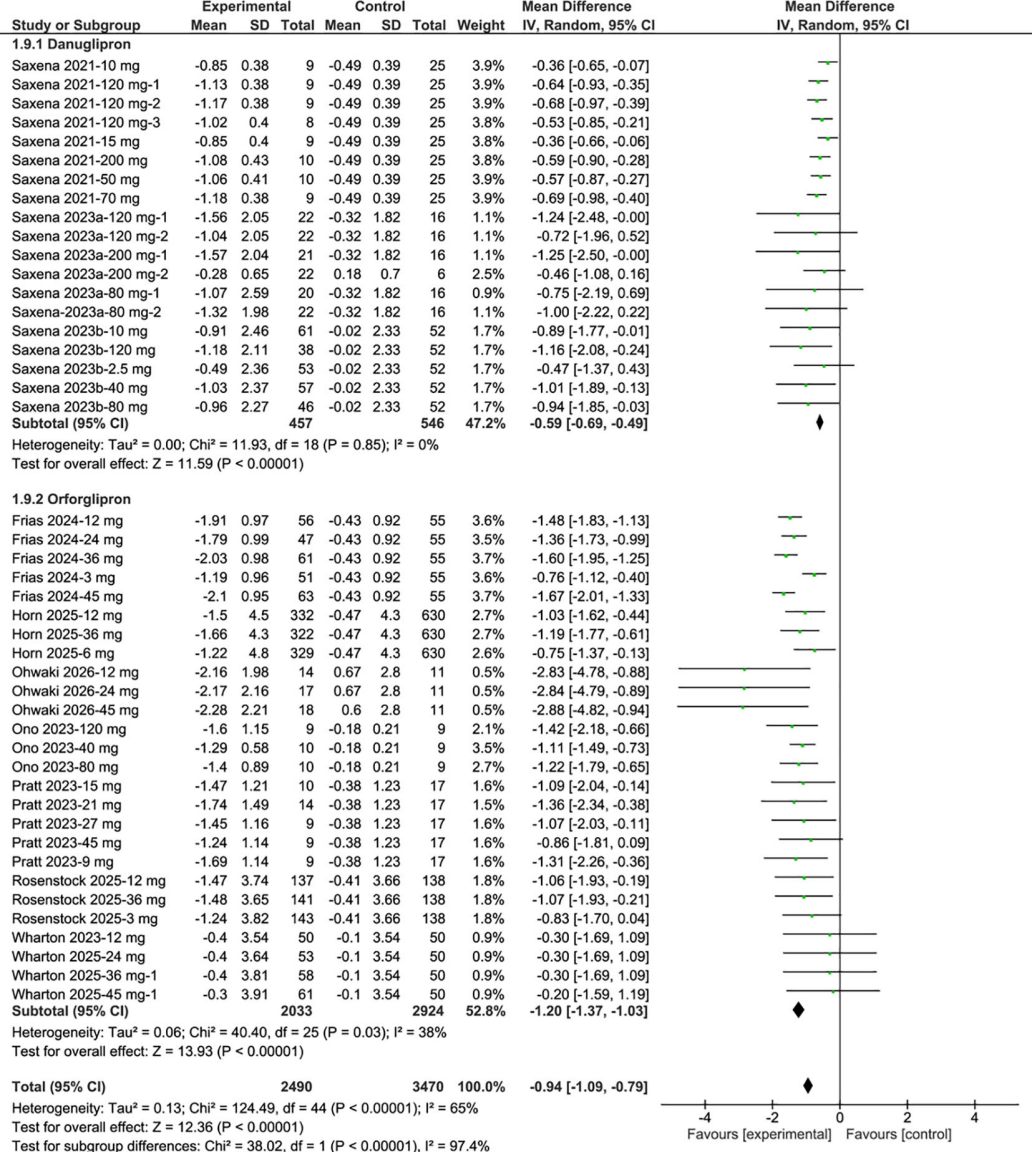
Forest plot of HbA1c (%). Forest plot showing the pooled effect of oral small-molecule GLP-1 receptor agonists on glycated hemoglobin (HbA1c) compared with control. Subgroup analyses were performed according to study drug (danuglipron and orforglipron).

Oral small-molecule GLP-1RAs significantly reduced HbA1c compared with control treatment (MD=-0.94, 95%CI: -1.09 to -0.79; P<0.00001; **Figure 7**), with substantial heterogeneity in the overall analysis (I² = 65%, heterogeneity P < 0.00001). In subgroup analyses, danuglipron reduced HbA1c (MD -0.59, 95%CI: -0.69 to -0.49; P<0.00001) without heterogeneity (I²=0%, heterogeneity P = 0.85), whereas orforglipron also reduced HbA1c (MD=-1.20, 95%CI: -1.37 to -1.03; P < 0.00001) with low-to-moderate heterogeneity (I² = 38%, heterogeneity P = 0.03). The magnitude of HbA1c reduction appeared greater in the orforglipron subgroup.

Visual inspection of the funnel plots for the main efficacy outcomes, including body weight change, ≥5%, ≥10%, and ≥15% body weight reduction, BMI, waist circumference, FBG, FINS, and HbA1c, suggested minimal selection bias (**Supplementary Figure 1**).

### TEAEs, AEs, and SAEs

**Figure 8:**
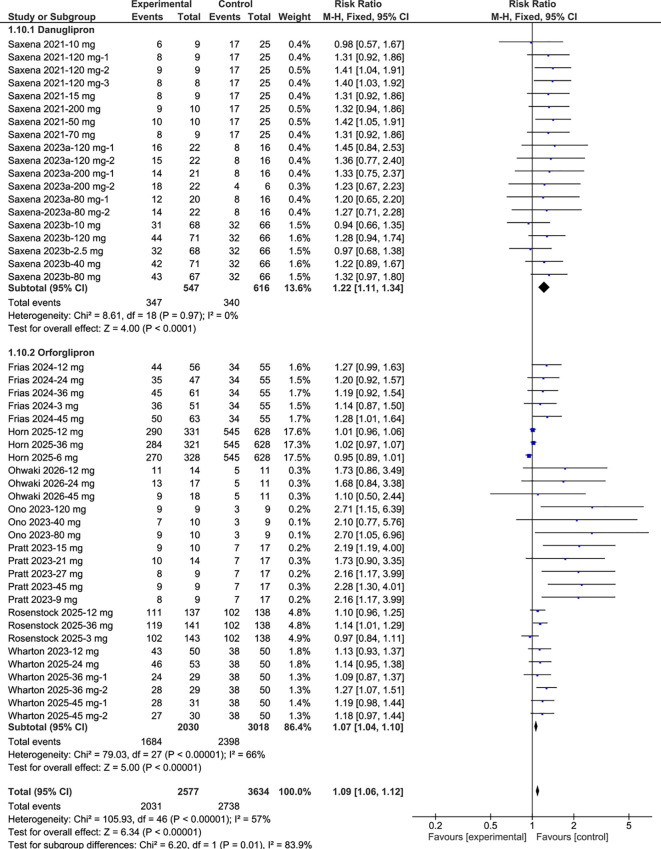
Forest plot of treatment-emergent adverse events (TEAEs). Forest plot showing the pooled risk ratio for TEAEs in participants receiving oral small-molecule GLP-1 receptor agonists versus control. Subgroup analyses were performed according to study drug.

Safety analyses showed that oral small-molecule GLP-1RAs were associated with increased risks of TEAEs and AEs, whereas no increase was observed for SAEs. For TEAEs, the pooled RR was 1.09 (95%CI: 1.06 to 1.12; P<0.00001; **Figure 8**), with heterogeneity (I²=57%, heterogeneity P < 0.00001). In subgroup analyses, both danuglipron (RR = 1.22, 95% CI: 1.11 to 1.34; P < 0.0001; I²=0%, heterogeneity P = 0.97) and orforglipron (RR = 1.07, 95% CI: 1.04 to 1.10; P < 0.00001; I² = 66%, heterogeneity P<0.00001) increased the risk of TEAEs, with a numerically greater effect for danuglipron.

**Figure 9:**
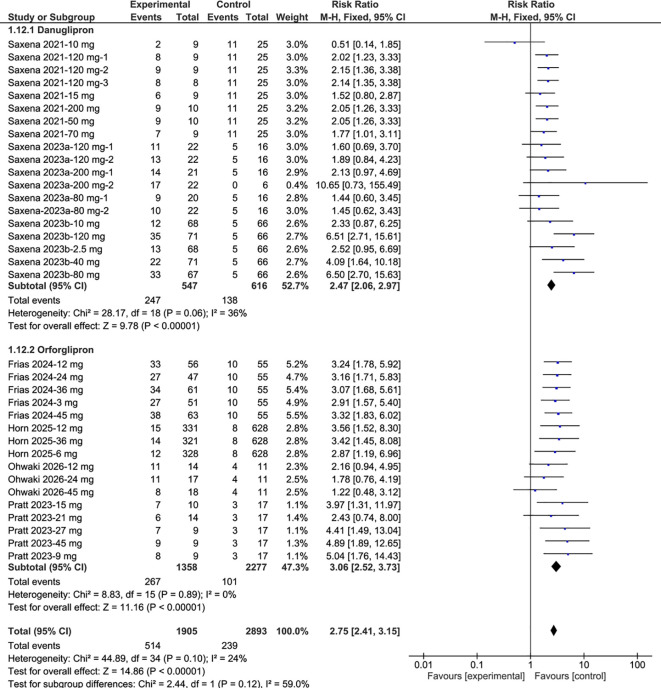
Forest plot of adverse events (AEs). Forest plot showing the pooled RR for overall AEs in participants treated with oral small-molecule GLP-1 receptor agonists compared with control. Subgroup analyses were performed according to study drug.

For AEs, the pooled RR was 2.75 (95%CI:2.41 to 3.15; P<0.00001; **Figure 9**), with low heterogeneity (I² = 24%, heterogeneity P = 0.10). Subgroup analyses also showed increased risks with both danuglipron (RR = 2.47, 95%CI:2.06 to 2.97; P<0.00001; I²=36%, heterogeneity P = 0.06) and orforglipron (RR = 3.06, 95%CI:2.52 to 3.73; P<0.00001; I²=0%, heterogeneity P = 0.89), with a numerically greater effect for orforglipron.

For SAEs, no significant increase was detected in the overall analysis (RR = 1.15, 95%CI:0.93 to 1.43; P = 0.20; **Supplementary Figure 2**), and heterogeneity was absent (I² = 0%, heterogeneity P = 0.78). This lack of significance was maintained in both subgroups: danuglipron (RR = 2.57, 95%CI:0.98 to 6.76; P = 0.06; I²=0%, heterogeneity P = 0.83) and orforglipron (RR = 1.09, 95%CI: 0.88 to 1.37; P = 0.42; I²=0%, heterogeneity P = 0.71).

### Gastrointestinal AEs

**Figure 10:**
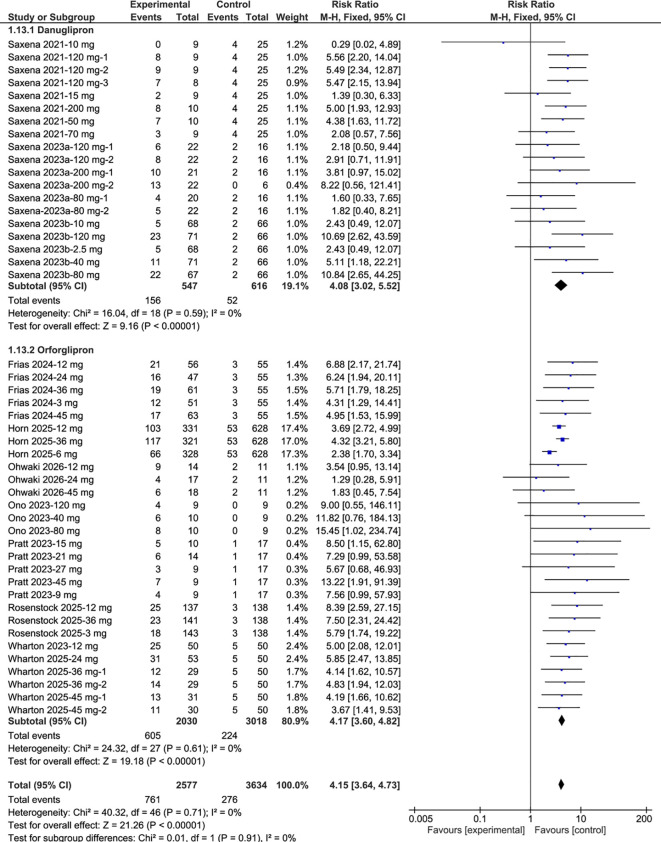
Forest plot of nausea. Forest plot showing the pooled RR for nausea associated with oral small-molecule GLP-1 receptor agonists compared with control, with subgroup analyses according to danuglipron and orforglipron.

Gastrointestinal AEs occurred more frequently in participants receiving oral small-molecule GLP-1RAs than in those receiving control treatment. For nausea, the pooled RR was 4.15 (95%CI: 3.64 to 4.73; P<0.00001; **Figure 10**), without heterogeneity (I² = 0%, heterogeneity P = 0.71). Both danuglipron (RR = 4.08, 95%CI: 3.02 to 5.52; P<0.00001; I²=0%, heterogeneity P = 0.59) and orforglipron (RR = 4.17, 95%CI:3.60 to 4.82; P<0.00001; I²=0%, heterogeneity P = 0.61) showed increased risk, with comparable effect sizes.

**Figure 11:**
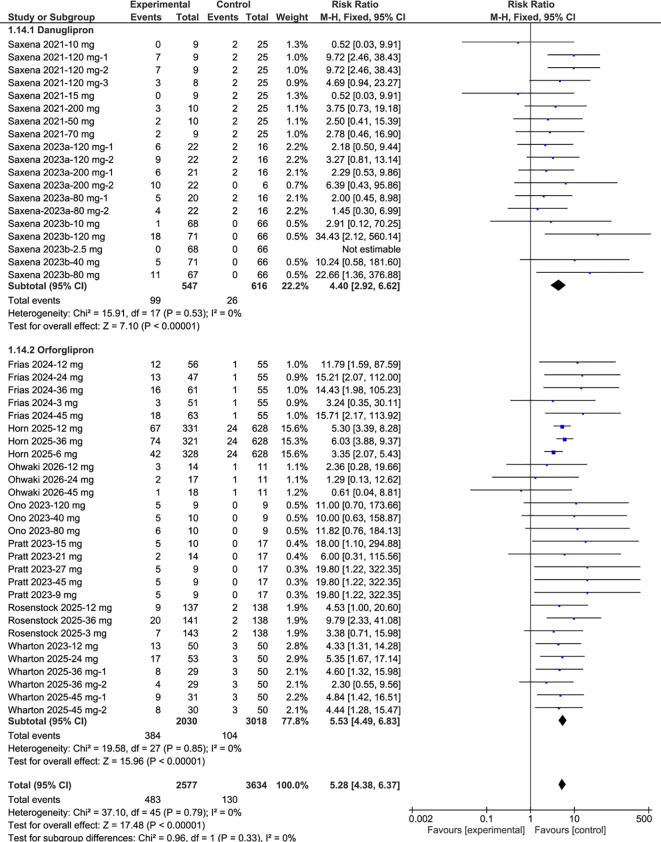
Forest plot of vomiting. Forest plot showing the pooled RR for vomiting associated with oral small-molecule GLP-1 receptor agonists compared with control, with subgroup analyses according to danuglipron and orforglipron.

For vomiting, the pooled RR was 5.28 (95%CI: 4.38 to 6.37; P<0.00001; **Figure 11**), without heterogeneity (I²=0%, heterogeneity P = 0.79). Significant increases were observed in both the danuglipron subgroup (RR = 4.40, 95%CI: 2.92 to 6.62; P<0.00001; I²=0%, heterogeneity P = 0.53) and orforglipron subgroup (RR = 5.53, 95%CI: 4.49 to 6.83; P<0.00001; I²=0%, heterogeneity P = 0.85), with a numerically larger effect for orforglipron.

**Figure 12:**
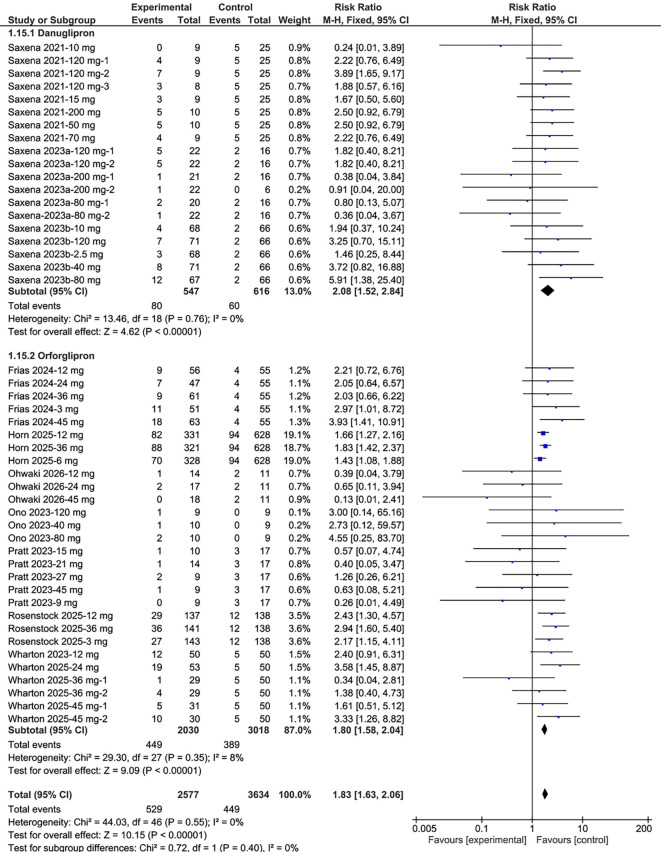
Forest plot of diarrhea. Forest plot showing the pooled RR for diarrhea associated with oral small-molecule GLP-1 receptor agonists compared with control, with subgroup analyses according to danuglipron and orforglipron.

For diarrhea, the pooled RR was 1.83 (95% CI:1.63 to 2.06; P<0.00001; **Figure 12**), without heterogeneity (I² = 0%, heterogeneity P = 0.55). Both danuglipron (RR = 2.08, 95%CI:1.52 to 2.84; P<0.00001; I²=0%, heterogeneity P = 0.76) and orforglipron (RR = 1.80, 95% CI: 1.58 to 2.04; P < 0.00001; I² = 8%, heterogeneity P = 0.35) were associated with increased risk, with a slightly larger effect in the danuglipron subgroup.

**Figure 13:**
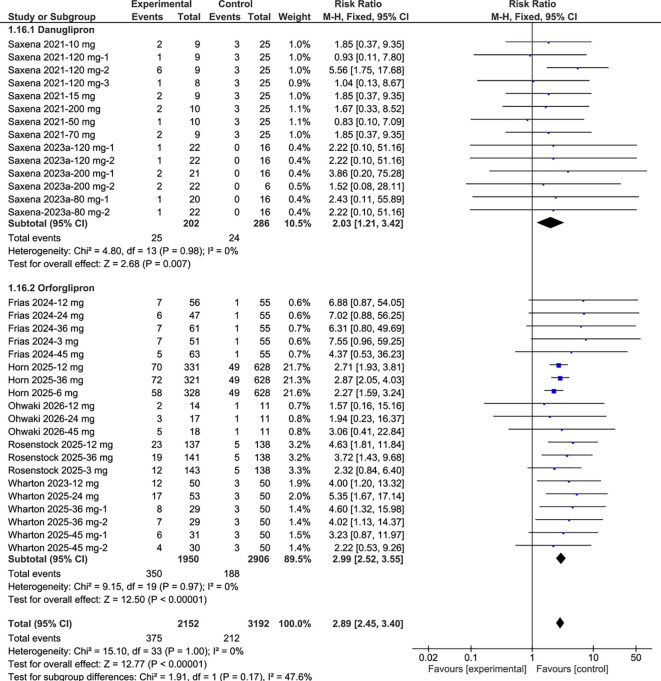
Forest plot of constipation. Forest plot showing the pooled RR for constipation associated with oral small-molecule GLP-1 receptor agonists compared with control, with subgroup analyses according to danuglipron and orforglipron.

For constipation, the pooled RR was 2.89 (95%CI:2.45 to 3.40; P<0.00001; **Figure 13**), again without heterogeneity (I² = 0%, heterogeneity P = 1.00). Both danuglipron (RR = 2.03, 95%CI:1.21 to 3.42; P = 0.007; I²=0%, heterogeneity P = 0.98) and orforglipron (RR = 2.99, 95%CI:2.52 to 3.55; P<0.00001; I²=0%, heterogeneity P = 0.97) increased constipation risk, with a numerically greater effect for orforglipron.

For dyspepsia, the pooled RR was 2.74 (95% CI: 2.28 to 3.29; P < 0.00001; **Supplementary Figure 3**), without heterogeneity (I²=0%, heterogeneity P = 0.98). Both danuglipron (RR = 3.16, 95%CI: 2.12 to 4.70; P<0.00001; I²=0%, heterogeneity P = 0.93) and orforglipron (RR = 2.64, 95% CI: 2.15 to 3.25; P<0.00001; I²=0%, heterogeneity P = 0.90) were significant, with a slightly greater effect estimate for danuglipron.

For decreased appetite, the pooled RR was 3.87 (95%CI: 3.06 to 4.89; P<0.00001; **Supplementary Figure 4**), with no heterogeneity (I² = 0%, heterogeneity P = 1.00). Significant increases were observed for danuglipron (RR = 6.07, 95%CI: 3.03 to 12.16; P<0.00001; I² = 0%, heterogeneity P = 0.95) and orforglipron (RR = 3.64, 95%CI: 2.84 to 4.67; P<0.00001; I²=0%, heterogeneity P = 1.00), with a numerically larger effect for danuglipron.

For eructation, the pooled RR was 11.62 (95%CI: 7.67 to 17.61; P<0.00001; **Supplementary Figure 5**), without heterogeneity (I² = 0%, heterogeneity P = 1.00). Both danuglipron (RR = 4.58, 95%CI: 1.24 to 16.83; P = 0.02; I²=0%, heterogeneity P = 0.93) and orforglipron (RR = 12.55, 95%CI: 8.08 to 19.49; P<0.00001; I²=0%, heterogeneity P = 1.00) increased the risk, with a markedly greater effect for orforglipron.

For gastro-esophageal reflux disease, the pooled RR was 3.09 (95%CI: 1.90 to 5.03; P<0.00001; **Supplementary Figure 6**), with no heterogeneity (I²=0%, heterogeneity P = 0.97). Significant increases were observed in both the danuglipron subgroup (RR = 2.44, 95%CI: 1.13 to 5.24; P = 0.02; I²=0%, heterogeneity P = 0.80) and the orforglipron subgroup (RR = 3.62, 95%CI: 1.92 to 6.83; P<0.0001; I²=0%, heterogeneity P = 0.98), with a numerically greater effect for orforglipron.

For abdominal distension, the pooled RR was 1.46 (95%CI:1.02 to 2.10; P = 0.04; **Supplementary Figure 7**), with low heterogeneity (I²=7%, heterogeneity P = 0.37). In subgroup analyses, danuglipron increased the risk (RR = 2.89, 95%CI:1.37 to 6.08; P = 0.005; I²=0%, heterogeneity P = 0.75), whereas orforglipron did not (RR = 1.15, 95%CI:0.75 to 1.75; P = 0.53; I²=19%, heterogeneity P = 0.28), suggesting that the overall increase was mainly driven by danuglipron.

For abdominal discomfort, the pooled RR was 7.87 (95% CI: 2.85 to 21.68; P < 0.0001; **Supplementary Figure 8**), with no heterogeneity (I²=0%, heterogeneity P = 1.00). Significant increases were observed with both danuglipron (RR = 11.63, 95%CI: 2.06 to 65.55; P = 0.005; I²=0%, heterogeneity P = 0.98) and orforglipron (RR = 6.73, 95%CI: 1.93 to 23.52; P = 0.003; I²=0%, heterogeneity P = 0.96), with a numerically greater effect for danuglipron.

For abdominal pain, the pooled RR was 2.02 (95%CI: 1.48 to 2.76; P<0.00001; **Supplementary Figure 9**), again without heterogeneity (I² = 0%, heterogeneity P = 0.99). In subgroup analyses, orforglipron increased risk of abdominal pain (RR = 1.98, 95%CI:1.44 to 2.71; P<0.0001; I²=0%, heterogeneity P = 0.97), whereas danuglipron did not reach statistical significance (RR = 3.35, 95% CI: 0.59 to 19.04; P = 0.17; I² = 0%, heterogeneity P = 0.96).

Visual inspection of the funnel plots for the analyzed adverse-event outcomes also suggested minimal selection bias (**Supplementary Figure 10**).

### Meta-regression analysis of body weight change and FBG

An exploratory meta-regression analysis was performed to examine whether dose contributed to the substantial heterogeneity observed for body weight change and FBG. Dose was not significantly associated with the effect size for body weight change (P = 0.913; **Supplementary Figure 11A**). Similarly, dose was not significantly associated with the effect size for FBG (P = 0.926; **Supplementary Figure 11B**).

## Discussion

This meta-analysis showed that oral small-molecule GLP-1RAs improved body weight, categorical weight-loss targets, BMI, waist circumference, FBG and HbA1c, while not significantly altering FINs. At the same time, these agents increased treatment-emergent adverse events, overall adverse events and a broad range of gastrointestinal symptoms, but did not increase SAEs. This overall pattern is consistent with the broader pharmacological profile of GLP-1 receptor agonism, in which meaningful metabolic efficacy is commonly accompanied by gastrointestinal tolerability limitations rather than major acute safety concerns (21–24). The present findings therefore place danuglipron and orforglipron firmly within the established incretin therapeutic class, while also supporting the feasibility of oral non-peptide GLP-1 receptor activation as an effective therapeutic strategy (22, 24).

The efficacy findings are mechanistically plausible. Activation of the GLP-1 receptor stimulates glucose-dependent insulin release, inhibits glucagon production, slows gastric emptying, and decreases appetite through coordinated pancreatic, gastrointestinal and central nervous system pathways (24). These integrated actions provide a biologically coherent explanation for reducing FBG and HbA1c, as well as the improvements in body weight, BMI and waist circumference observed in this analysis (24). The marked increase in the proportions of participants achieving ≥5%, ≥10% and ≥15% body-weight reduction is also biologically credible, as sustained appetite suppression and delayed gastric emptying reduce energy intake over time and thereby promote progressive anthropometric improvement. By contrast, the absence of a significant pooled effect on FINs is not unexpected, because GLP-1RAs do not stimulate insulin secretion constitutively, but instead amplify insulin release in a glucose-dependent manner (25).

Our findings are broadly consistent with previous studies of peptide-based GLP-1RAs, although such comparisons remain indirect. Injectable liraglutide and semaglutide have already demonstrated clinically meaningful weight reduction in obesity, and semaglutide 2.4 mg substantially increased the proportion of patients achieving higher categorical weight-loss thresholds in the STEP program (5, 26–28). More recently, tirzepatide showed even greater reductions in body weight and glycemic indices in obesity and T2D trials (4, 29, 30). Against this background, the efficacy of oral small-molecule GLP-1RAs in our pooled analysis appears promising, particularly because significant benefits were observed across both anthropometric and glycemic domains while avoiding injection (5, 26, 29). Nevertheless, the magnitude of benefit should not be interpreted as equivalent or superior on the basis of cross-trial comparison alone, because treatment duration, titration schedules, background therapy and enrolled populations differed substantially among studies (29, 30). Therefore, comparisons with injectable GLP-1RAs or other incretin-based therapies should be regarded as indirect and hypothesis-generating, and direct head-to-head trials are needed to determine their relative efficacy, tolerability, and long-term benefit–risk profiles. The pharmacokinetic properties of oral small-molecule GLP-1RAs may also influence their clinical performance. Differences in half-life and metabolic stability can affect dosing frequency, exposure consistency, dose escalation, gastrointestinal tolerability, and hepatic safety monitoring. Orforglipron has been reported to have a relatively long half-life, supporting once-daily oral dosing, whereas danuglipron has been evaluated using different dosing and escalation strategies (9, 11, 13). Therefore, future studies should compare the half-life, metabolic stability, exposure–response relationships, and hepatic safety profiles of these agents to better define their benefit–risk balance.

The safety findings are also mechanistically coherent and align with prior reports of GLP-1RA tolerability. Nausea, vomiting, diarrhea, constipation, dyspepsia, decreased appetite, eructation, reflux-related symptoms and abdominal complaints were all increased in our pooled analyses, and this constellation closely resembles the adverse-event profile reported across the broader GLP-1RA literature (31). Delayed gastric emptying is likely central to this toxicity pattern. Although therapeutically advantageous for lowering postprandial glucose excursions and enhancing satiety, it can also promote gastric retention and upper gastrointestinal symptoms (31). In addition, central pathways involved in appetite suppression overlap with nausea-related neurocircuitry, which may explain why decreased appetite and gastrointestinal intolerance frequently coexist during treatment. Importantly, although TEAEs and overall AEs increased, SAEs did not, suggesting that the principal clinical trade-off with these agents is tolerability rather than serious short-term toxicity (32).

From a clinical perspective, the major potential advantage of oral small-molecule GLP-1RAs may lie in route of administration rather than in a fundamentally different efficacy–safety balance. Peptide-based GLP-1 receptor agonists have already established the class as an effective treatment for obesity and T2D, with additional cardiovascular and renal benefits demonstrated in large outcome trials and meta-analyses (33–37). Oral small molecules may broaden acceptance and accessibility by avoiding injections and, unlike oral semaglutide, may also avoid stringent fasting-related administration requirements (38, 39). However, our pooled data suggest that these compounds do not eliminate the class-associated gastrointestinal tolerability burden. Their principal value may therefore be to expand therapeutic options within the incretin field, rather than to overcome the core gastrointestinal liabilities of GLP-1-based treatment (40).

Evidence from established GLP-1RAs has shown reductions in major adverse cardiovascular events, cardiovascular mortality, all-cause mortality and composite kidney outcomes in patients with T2D and high cardiovascular risk (32–38). In addition, semaglutide has demonstrated cardiovascular benefit in patients with overweight or obesity and established cardiovascular disease but without diabetes, suggesting that the cardiovascular benefit of GLP-1 receptor activation may extend beyond glucose lowering alone (39). Renal protection may be mediated through improved glycemic control, weight reduction, blood pressure lowering, reduced albuminuria, and anti-inflammatory or endothelial effects (32–38). However, direct cardiovascular and renal outcome data for oral small-molecule GLP-1RAs such as danuglipron and orforglipron remain limited, as currently available trials mainly focus on glycemic, anthropometric and short-term safety outcomes rather than hard organ-protective endpoints (7–18, 40, 41). Therefore, although these agents may share the broader cardiovascular and renal protective potential of the GLP-1RA class, dedicated long-term cardiovascular and renal outcome trials are still needed to determine whether they reduce major adverse cardiovascular events, heart failure hospitalization, sustained eGFR decline, kidney failure, or renal death.

Several limitations should be acknowledged. First, the number of included trials remained limited for several subgroup analyses and for some less frequent adverse events, reducing precision for certain pooled estimates. Second, although leave-one-out sensitivity analyses and exploratory dose-based meta-regression were performed, the sources of heterogeneity for body weight change and FBG could not be fully identified. Dose was not a significant moderator for either outcome, and no single study accounted for the observed heterogeneity. Therefore, residual heterogeneity may be attributable to multiple interacting factors, including differences in study population, trial phase, treatment duration, dose-escalation regimen, target dose, baseline metabolic status, background therapy, and outcome measurement. Given the limited number of included studies and multiple treatment arms sharing common comparator groups, the meta-regression findings should be considered exploratory, and the pooled estimates for highly heterogeneous outcomes should be interpreted with caution. Visual inspection of the funnel plots for the main efficacy outcomes did not show obvious asymmetry; however, these findings should be interpreted cautiously because the number of included studies was limited, and funnel plot assessment has low sensitivity when fewer than 10 studies are available for an outcome. Third, most available data were derived from sponsor-led randomized trials rather than independent pragmatic or real-world studies. Fourth, follow-up was relatively short in several included trials, limiting inference regarding long-term durability of weight loss, sustained glycemic control and uncommon adverse events. Fifth, comparisons with peptide-based GLP-1RAs in this discussion are indirect and should not be overinterpreted in the absence of robust head-to-head evidence. Finally, hepatic safety should also be noted. Although lotiglipron was not included in the present meta-analysis (41), Pfizer discontinued its development in 2023 after elevated transaminases were observed in clinical studies, indicating that liver enzyme abnormalities may be a compound-specific safety concern among oral small-molecule GLP-1RAs. In the included danuglipron and orforglipron trials, gastrointestinal intolerance was the dominant adverse-event signal and SAEs were not significantly increased; however, uncommon hepatic events may not be fully captured in short-term trials. Future studies should therefore include systematic assessment and transparent reporting of hepatic safety outcomes, including liver-function abnormalities and liver-related treatment discontinuations.

## Conclusion

Oral small-molecule GLP-1RAs provide meaningful benefits in weight reduction and glycemic control in patients with T2D and obesity, supporting their promise as effective oral incretin therapies. However, their use is associated with increased gastrointestinal AEs, indicating that the overall clinical value of these agents will depend on achieving an appropriate balance between efficacy and tolerability. Longer-term and head-to-head studies are warranted to better define their place in therapy.

## Data Availability

The original contributions presented in the study are included in the article/**Supplementary Material**. Further inquiries can be directed to the corresponding author.
