# Supplementary material for: Investigation into the efficacy and safety profile of oral small-molecule GLP-1 receptor agonists in type 2 diabetes and obesity: a systematic review and meta-analysis
Source: Front Endocrinol (Lausanne). 2026 Jul 7;17:1854779. doi: 10.3389/fendo.2026.1854779 (PMC13385192; doi:10.3389/fendo.2026.1854779)
Supplement: Supplementary file 1 [file DataSheet1.pdf]

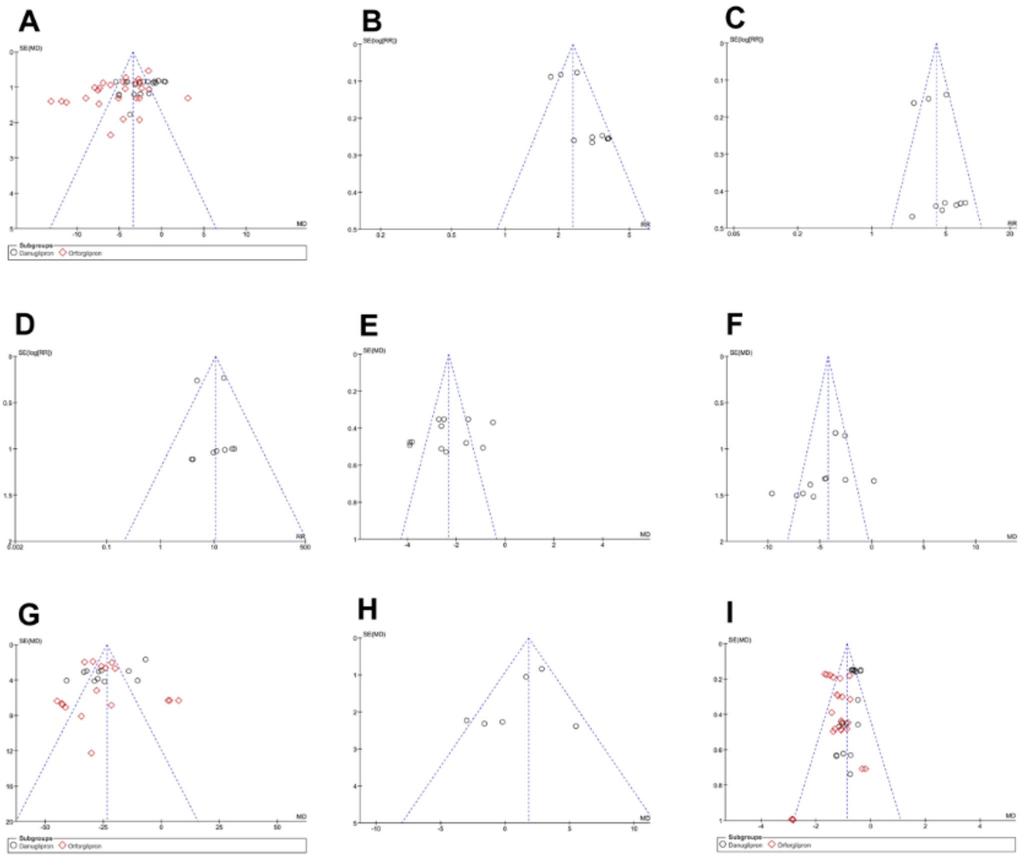

Supplemental Figure S1. Funnel plots for the main efficacy outcomes. Funnel plots assessing potential publication bias for the principal efficacy outcomes, including (A) body weight change, (B) ≥5%, (C) ≥10%, and (D) ≥15% body weight reduction, (E) body mass index, (F) waist circumference, (G) fasting blood glucose, (H) fasting insulin, and (I) HbA1c.

Study or Subgroup	Experimental Events	Experimental Total	Control Events	Control Total	Weight	Risk Ratio M-H, Fixed, 95% CI
**1.11.1 Danuglipron**						
Saxena 2021-10 mg	0	9	0	25		Not estimable
Saxena 2021-120 mg-1	0	9	0	25		Not estimable
Saxena 2021-120 mg-2	1	9	0	25	0.2%	7.80 [0.35, 176.01]
Saxena 2021-120 mg-3	0	8	0	25		Not estimable
Saxena 2021-15 mg	0	9	0	25		Not estimable
Saxena 2021-200 mg	0	10	0	25		Not estimable
Saxena 2021-50 mg	0	10	0	25		Not estimable
Saxena 2021-70 mg	0	9	0	25		Not estimable
Saxena 2023a-120 mg-1	0	22	0	16		Not estimable
Saxena 2023a-120 mg-2	0	22	0	16		Not estimable
Saxena 2023a-200 mg-1	0	21	0	16		Not estimable
Saxena 2023a-200 mg-2	0	22	0	6		Not estimable
Saxena 2023a-80 mg-1	0	20	0	16		Not estimable
Saxena-2023a-80 mg-2	0	22	0	16		Not estimable
Saxena 2023b-10 mg	2	68	1	66	0.7%	1.94 [0.18, 20.90]
Saxena 2023b-120 mg	1	71	1	66	0.7%	0.93 [0.06, 14.56]
Saxena 2023b-2.5 mg	1	68	1	66	0.7%	0.97 [0.06, 15.20]
Saxena 2023b-40 mg	6	71	1	66	0.7%	5.58 [0.69, 45.11]
Saxena 2023b-80 mg	2	67	1	66	0.7%	1.97 [0.18, 21.21]
**Subtotal (95% CI)**		**547**		**616**	**3.8%**	**2.57 [0.98, 6.76]**
Total events	13		5			
Heterogeneity: Chi² = 2.12, df = 5 (P = 0.83); I² = 0%						
Test for overall effect: Z = 1.91 (P = 0.06)						
**1.11.2 Orforglipron**						
Frias 2024-12 mg	1	56	3	55	2.2%	0.33 [0.04, 3.05]
Frias 2024-24 mg	5	47	3	55	2.0%	1.95 [0.49, 7.73]
Frias 2024-36 mg	2	61	3	55	2.2%	0.60 [0.10, 3.46]
Frias 2024-3 mg	3	51	3	55	2.1%	1.08 [0.23, 5.10]
Frias 2024-45 mg	1	63	3	55	2.3%	0.29 [0.03, 2.72]
Horn 2025-12 mg	34	331	55	628	27.1%	1.17 [0.78, 1.76]
Horn 2025-36 mg	35	321	55	628	26.5%	1.24 [0.83, 1.86]
Horn 2025-6 mg	24	328	55	628	26.9%	0.84 [0.53, 1.32]
Ohwaki 2026-12 mg	0	14	0	11		Not estimable
Ohwaki 2026-24 mg	0	17	0	11		Not estimable
Ohwaki 2026-45 mg	0	18	0	11		Not estimable
Ono 2023-120 mg	0	9	0	9		Not estimable
Ono 2023-40 mg	0	10	0	9		Not estimable
Ono 2023-80 mg	0	10	0	9		Not estimable
Pratt 2023-15 mg	0	10	1	17	0.8%	0.55 [0.02, 12.25]
Pratt 2023-21 mg	0	14	1	17	1.0%	0.40 [0.02, 9.12]
Pratt 2023-27 mg	0	9	1	17	0.8%	0.60 [0.03, 13.40]
Pratt 2023-45 mg	0	9	1	17	0.8%	0.60 [0.03, 13.40]
Pratt 2023-9 mg	0	9	1	17	0.8%	0.60 [0.03, 13.40]
Rosenstock 2025-12 mg	0	137	0	138		Not estimable
Rosenstock 2025-36 mg	0	141	0	138		Not estimable
Rosenstock 2025-3 mg	0	143	0	138		Not estimable
Wharton 2023-12 mg	0	50	0	50		Not estimable
Wharton 2025-24 mg	2	53	0	50	0.4%	4.72 [0.23, 96.01]
Wharton 2025-36 mg-1	0	29	0	50		Not estimable
Wharton 2025-36 mg-2	2	29	0	50	0.3%	8.50 [0.42, 171.19]
Wharton 2025-45 mg-1	3	31	0	50	0.3%	11.16 [0.60, 208.94]
Wharton 2025-45 mg-2	0	30	0	50		Not estimable
**Subtotal (95% CI)**		**2030**		**3018**	**96.2%**	**1.09 [0.88, 1.37]**
Total events	112		185			
Heterogeneity: Chi² = 11.56, df = 15 (P = 0.71); I² = 0%						
Test for overall effect: Z = 0.80 (P = 0.42)						
**Total (95% CI)**		**2577**		**3634**	**100.0%**	**1.15 [0.93, 1.43]**
Total events	125		190			
Heterogeneity: Chi² = 15.87, df = 21 (P = 0.78); I² = 0%						
Test for overall effect: Z = 1.28 (P = 0.20)						
Test for subgroup differences: Chi² = 2.85, df = 1 (P = 0.09), I² = 64.9%						

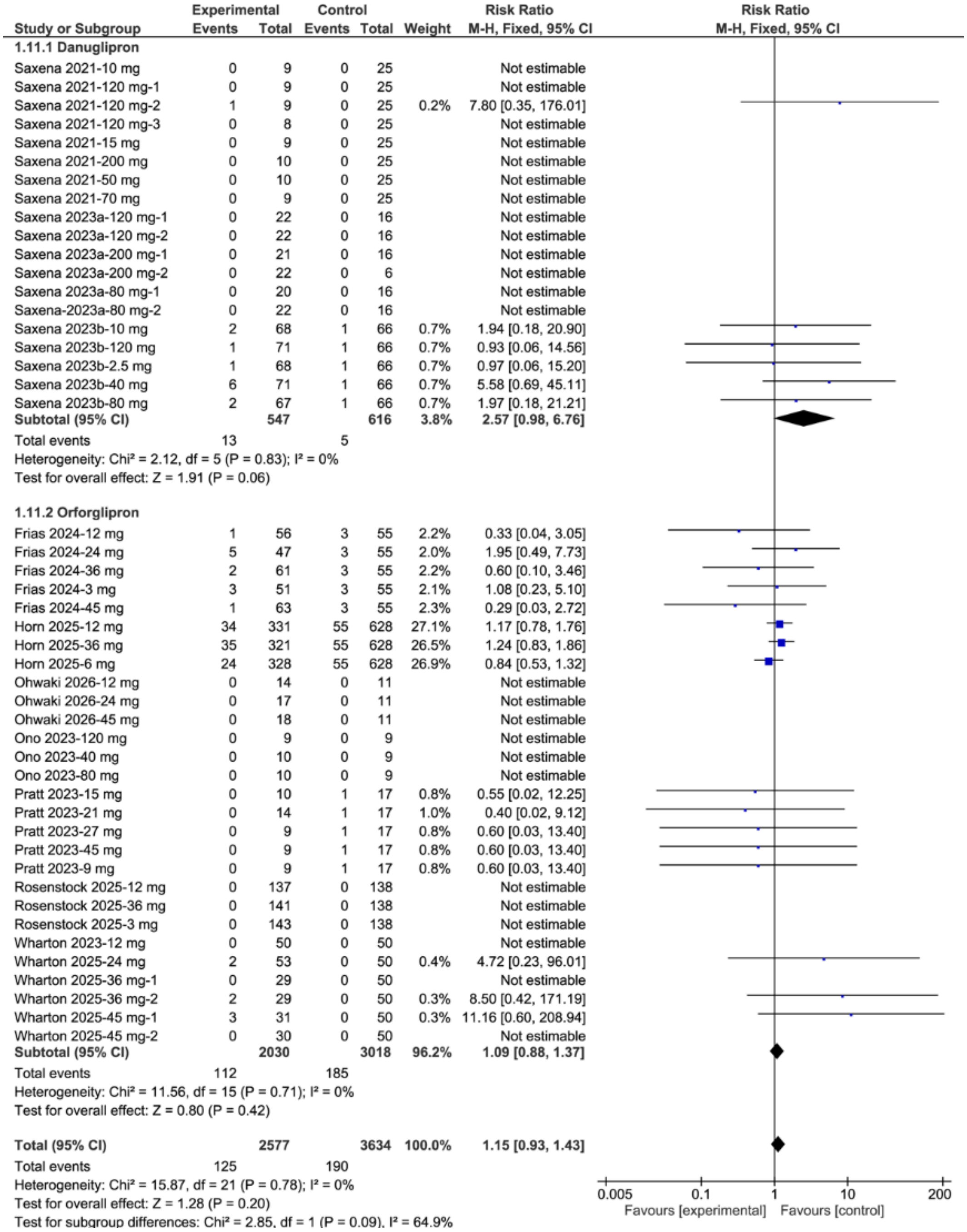

Supplemental Figure S2. Forest plot of serious adverse events (SAEs). Forest plot showing the pooled RR for SAEs associated with oral small-molecule GLP-1 receptor agonists compared with control, with subgroup analyses according to study drug.

Study or Subgroup	Experimental Events	Experimental Total	Control Events	Control Total	Weight	Risk Ratio M-H, Fixed, 95% CI
**1.18.1 Danuglipron**						
Saxena 2021-10 mg	0	9	4	25	2.0%	0.29 [0.02, 4.89]
Saxena 2021-120 mg-1	4	9	4	25	1.7%	2.78 [0.87, 8.84]
Saxena 2021-120 mg-2	7	9	4	25	1.7%	4.86 [1.85, 12.74]
Saxena 2021-120 mg-3	3	8	4	25	1.5%	2.34 [0.66, 8.33]
Saxena 2021-15 mg	0	9	4	25	2.0%	0.29 [0.02, 4.89]
Saxena 2021-200 mg	5	10	4	25	1.8%	3.13 [1.05, 9.31]
Saxena 2021-50 mg	5	10	4	25	1.8%	3.13 [1.05, 9.31]
Saxena 2021-70 mg	4	9	4	25	1.7%	2.78 [0.87, 8.84]
Saxena 2023a-120 mg-1	2	22	0	16	0.5%	3.70 [0.19, 72.10]
Saxena 2023a-120 mg-2	5	22	0	16	0.5%	8.13 [0.48, 137.27]
Saxena 2023a-200 mg-1	1	21	0	16	0.4%	2.32 [0.10, 53.42]
Saxena 2023a-200 mg-2	1	22	0	6	0.6%	0.91 [0.04, 20.00]
Saxena 2023a-80 mg-1	1	20	0	16	0.4%	2.43 [0.11, 55.89]
Saxena-2023a-80 mg-2	1	22	0	16	0.5%	2.22 [0.10, 51.16]
Saxena 2023b-10 mg	3	68	0	66	0.4%	6.80 [0.36, 129.10]
Saxena 2023b-120 mg	2	71	0	66	0.4%	4.65 [0.23, 95.16]
Saxena 2023b-2.5 mg	4	68	0	66	0.4%	8.74 [0.48, 159.20]
Saxena 2023b-40 mg	2	71	0	66	0.4%	4.65 [0.23, 95.16]
Saxena 2023b-80 mg	9	67	0	66	0.4%	18.72 [1.11, 315.25]
**Subtotal (95% CI)**		**547**		**616**	**18.9%**	**3.16 [2.12, 4.70]**
Total events	59		32			
Heterogeneity: Chi² = 10.13, df = 18 (P = 0.93); I² = 0%						
Test for overall effect: Z = 5.66 (P < 0.00001)						
**1.18.2 Orforglipron**						
Frias 2024-12 mg	4	56	2	55	1.6%	1.96 [0.37, 10.29]
Frias 2024-24 mg	3	47	2	55	1.4%	1.76 [0.31, 10.06]
Frias 2024-36 mg	5	61	2	55	1.7%	2.25 [0.46, 11.15]
Frias 2024-3 mg	6	51	2	55	1.5%	3.24 [0.68, 15.31]
Frias 2024-45 mg	4	63	2	55	1.7%	1.75 [0.33, 9.17]
Horn 2025-12 mg	51	331	22	628	11.9%	4.40 [2.72, 7.12]
Horn 2025-36 mg	35	321	22	628	11.7%	3.11 [1.86, 5.21]
Horn 2025-6 mg	30	328	22	628	11.8%	2.61 [1.53, 4.45]
Ohwaki 2026-12 mg	1	14	0	11	0.4%	2.40 [0.11, 53.77]
Ohwaki 2026-24 mg	1	17	0	11	0.5%	2.00 [0.09, 45.12]
Ohwaki 2026-45 mg	1	18	0	11	0.5%	1.89 [0.08, 42.82]
Pratt 2023-15 mg	1	10	1	17	0.6%	1.70 [0.12, 24.29]
Pratt 2023-21 mg	0	14	1	17	1.1%	0.40 [0.02, 9.12]
Pratt 2023-27 mg	2	9	1	17	0.5%	3.78 [0.39, 36.22]
Pratt 2023-45 mg	3	9	1	17	0.5%	5.67 [0.68, 46.93]
Pratt 2023-9 mg	2	9	1	17	0.5%	3.78 [0.39, 36.22]
Rosenstock 2025-12 mg	28	137	9	138	7.0%	3.13 [1.54, 6.39]
Rosenstock 2025-36 mg	21	141	9	138	7.1%	2.28 [1.08, 4.81]
Rosenstock 2025-3 mg	15	143	9	138	7.2%	1.61 [0.73, 3.55]
Wharton 2023-12 mg	8	50	3	50	2.4%	2.67 [0.75, 9.47]
Wharton 2025-24 mg	4	53	3	50	2.4%	1.26 [0.30, 5.34]
Wharton 2025-36 mg-1	1	29	3	50	1.7%	0.57 [0.06, 5.27]
Wharton 2025-36 mg-2	1	29	3	50	1.7%	0.57 [0.06, 5.27]
Wharton 2025-45 mg-1	3	31	3	50	1.8%	1.61 [0.35, 7.50]
Wharton 2025-45 mg-2	2	30	3	50	1.8%	1.11 [0.20, 6.27]
**Subtotal (95% CI)**		**2001**		**2991**	**81.1%**	**2.64 [2.15, 3.25]**
Total events	232		126			
Heterogeneity: Chi² = 15.52, df = 24 (P = 0.90); I² = 0%						
Test for overall effect: Z = 9.18 (P < 0.00001)						
**Total (95% CI)**		**2548**		**3607**	**100.0%**	**2.74 [2.28, 3.29]**
Total events	291		158			
Heterogeneity: Chi² = 26.09, df = 43 (P = 0.98); I² = 0%						
Test for overall effect: Z = 10.74 (P < 0.00001)						
Test for subgroup differences: Chi² = 0.60, df = 1 (P = 0.44), I² = 0%						

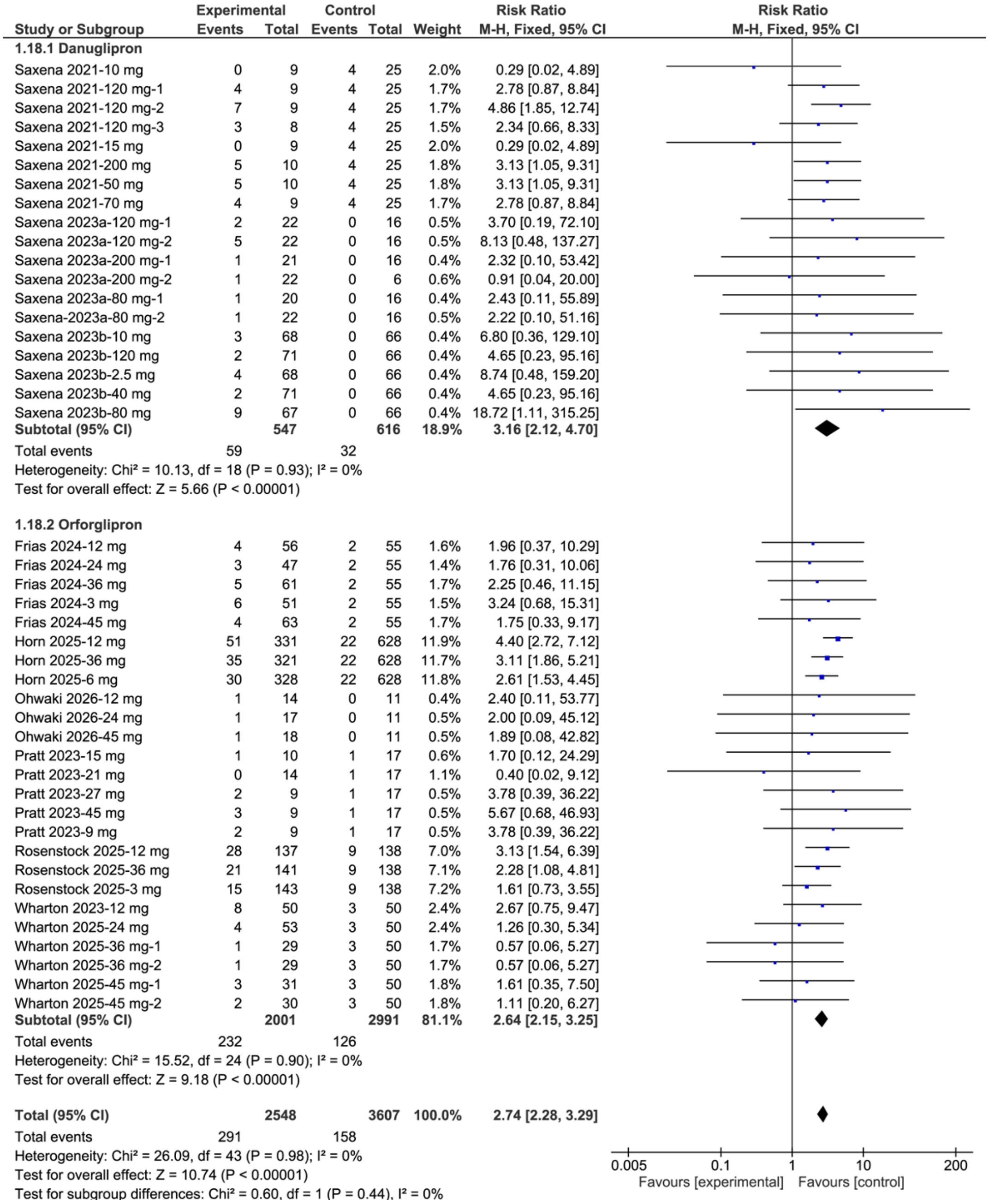

Supplemental Figure S3. Forest plot of dyspepsia. Forest plot showing the pooled RR for dyspepsia associated with oral small-molecule GLP-1 receptor agonists compared with control, with subgroup analyses according to danuglipron and orforglipron.

Study or Subgroup	Experimental Events	Experimental Total	Control Events	Control Total	Weight	Risk Ratio M-H, Fixed, 95% CI
**1.17.1 Danuglipron**						
Saxena 2021-10 mg	0	9	1	25	1.2%	0.87 [0.04, 19.56]
Saxena 2021-120 mg-1	6	9	1	25	0.8%	16.67 [2.31, 120.13]
Saxena 2021-120 mg-2	2	9	1	25	0.8%	5.56 [0.57, 54.12]
Saxena 2021-120 mg-3	3	8	1	25	0.7%	9.38 [1.13, 77.99]
Saxena 2021-15 mg	1	9	1	25	0.8%	2.78 [0.19, 39.91]
Saxena 2021-200 mg	5	10	1	25	0.8%	12.50 [1.66, 94.03]
Saxena 2021-50 mg	1	10	1	25	0.8%	2.50 [0.17, 36.21]
Saxena 2021-70 mg	1	9	1	25	0.8%	2.78 [0.19, 39.91]
Saxena 2023b-10 mg	0	68	0	66		Not estimable
Saxena 2023b-120 mg	5	71	0	66	0.7%	10.24 [0.58, 181.60]
Saxena 2023b-2.5 mg	2	68	0	66	0.7%	4.86 [0.24, 99.26]
Saxena 2023b-40 mg	2	71	0	66	0.7%	4.65 [0.23, 95.16]
Saxena 2023b-80 mg	1	67	0	66	0.7%	2.96 [0.12, 71.28]
**Subtotal (95% CI)**		**418**		**530**	**9.5%**	**6.07 [3.03, 12.16]**
Total events	29		8			
Heterogeneity: Chi² = 4.62, df = 11 (P = 0.95); I² = 0%						
Test for overall effect: Z = 5.09 (P < 0.00001)						
**1.17.2 Orforglipron**						
Horn 2025-12 mg	30	331	18	628	17.7%	3.16 [1.79, 5.59]
Horn 2025-36 mg	49	321	18	628	17.4%	5.33 [3.16, 8.99]
Horn 2025-6 mg	27	328	18	628	17.6%	2.87 [1.61, 5.14]
Ohwaki 2026-12 mg	5	14	1	11	1.6%	3.93 [0.53, 28.93]
Ohwaki 2026-24 mg	3	17	1	11	1.7%	1.94 [0.23, 16.37]
Ohwaki 2026-45 mg	3	18	1	11	1.8%	1.83 [0.22, 15.51]
Ono 2023-120 mg	1	9	0	9	0.7%	3.00 [0.14, 65.16]
Ono 2023-40 mg	0	10	0	9		Not estimable
Ono 2023-80 mg	1	10	0	9	0.7%	2.73 [0.12, 59.57]
Pratt 2023-15 mg	4	10	2	17	2.1%	3.40 [0.75, 15.34]
Pratt 2023-21 mg	4	14	2	17	2.6%	2.43 [0.52, 11.36]
Pratt 2023-27 mg	5	9	2	17	2.0%	4.72 [1.13, 19.67]
Pratt 2023-45 mg	6	9	2	17	2.0%	5.67 [1.42, 22.56]
Pratt 2023-9 mg	4	9	2	17	2.0%	3.78 [0.85, 16.81]
Rosenstock 2025-12 mg	5	137	3	138	4.3%	1.68 [0.41, 6.89]
Rosenstock 2025-36 mg	14	141	3	138	4.3%	4.57 [1.34, 15.54]
Rosenstock 2025-3 mg	16	143	3	138	4.4%	5.15 [1.53, 17.27]
Wharton 2023-12 mg	4	50	1	50	1.4%	4.00 [0.46, 34.54]
Wharton 2025-24 mg	4	53	1	50	1.5%	3.77 [0.44, 32.62]
Wharton 2025-36 mg-1	0	29	1	50	1.6%	0.57 [0.02, 13.47]
Wharton 2025-36 mg-2	1	29	1	50	1.0%	1.72 [0.11, 26.54]
Wharton 2025-45 mg-1	3	31	1	50	1.1%	4.84 [0.53, 44.48]
Wharton 2025-45 mg-2	2	30	1	50	1.1%	3.33 [0.32, 35.21]
**Subtotal (95% CI)**		**1752**		**2743**	**90.5%**	**3.64 [2.84, 4.67]**
Total events	191		82			
Heterogeneity: Chi² = 7.78, df = 21 (P = 1.00); I² = 0%						
Test for overall effect: Z = 10.16 (P < 0.00001)						
**Total (95% CI)**		**2170**		**3273**	**100.0%**	**3.87 [3.06, 4.89]**
Total events	220		90			
Heterogeneity: Chi² = 13.83, df = 33 (P = 1.00); I² = 0%						
Test for overall effect: Z = 11.35 (P < 0.00001)						
Test for subgroup differences: Chi² = 1.84, df = 1 (P = 0.18), I² = 45.5%						

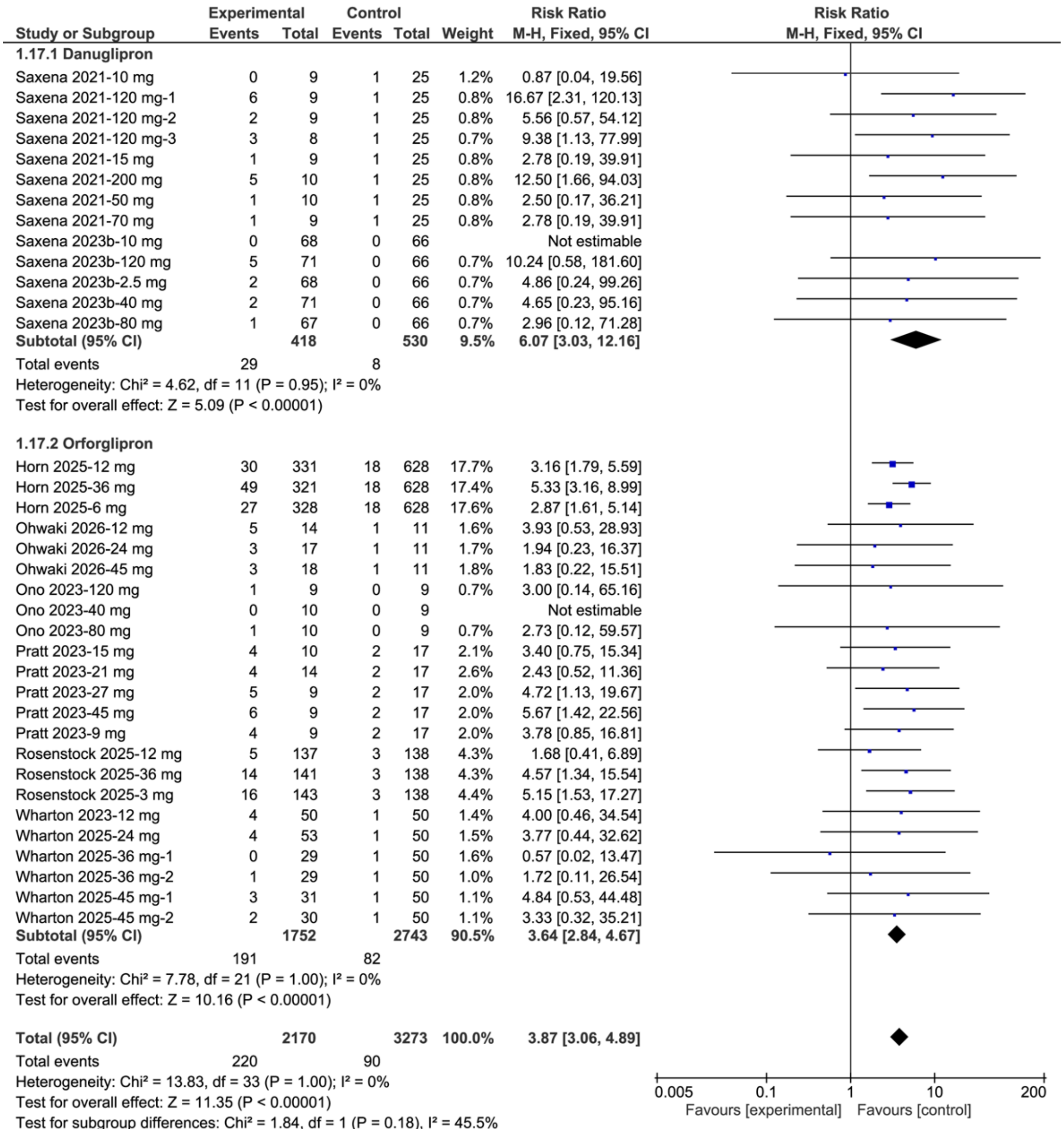

Supplemental Figure S4. Forest plot of decreased appetite. Forest plot showing the pooled RR for decreased appetite associated with oral small-molecule GLP-1 receptor agonists compared with control, with subgroup analyses according to danuglipron and orforglipron.

Study or Subgroup	Experimental Events	Experimental Total	Control Events	Control Total	Weight	Risk Ratio M-H, Fixed, 95% CI
**1.20.1 Danuglipron**						
Saxena 2021-10 mg	0	9	0	25		Not estimable
Saxena 2021-120 mg-1	1	9	0	25	1.4%	7.80 [0.35, 176.01]
Saxena 2021-120 mg-2	0	9	0	25		Not estimable
Saxena 2021-120 mg-3	0	8	0	25		Not estimable
Saxena 2021-15 mg	0	9	0	25		Not estimable
Saxena 2021-200 mg	0	10	0	25		Not estimable
Saxena 2021-50 mg	2	10	0	25	1.5%	11.82 [0.62, 226.57]
Saxena 2021-70 mg	0	9	0	25		Not estimable
Saxena 2023a-120 mg-1	0	22	0	16		Not estimable
Saxena 2023a-120 mg-2	1	22	0	16	2.9%	2.22 [0.10, 51.16]
Saxena 2023a-200 mg-1	0	21	0	16		Not estimable
Saxena 2023a-200 mg-2	0	22	0	6		Not estimable
Saxena 2023a-80 mg-1	1	20	0	16	2.8%	2.43 [0.11, 55.89]
Saxena-2023a-80 mg-2	2	22	0	16	2.9%	3.70 [0.19, 72.10]
**Subtotal (95% CI)**		**202**		**286**	**11.6%**	**4.58 [1.24, 16.83]**
Total events	7		0			
Heterogeneity: Chi² = 0.89, df = 4 (P = 0.93); I² = 0%						
Test for overall effect: Z = 2.29 (P = 0.02)						
**1.20.2 Orforglipron**						
Frias 2024-12 mg	2	56	0	55	2.6%	4.91 [0.24, 100.05]
Frias 2024-24 mg	3	47	0	55	2.3%	8.17 [0.43, 154.17]
Frias 2024-36 mg	8	61	0	55	2.7%	15.35 [0.91, 259.98]
Frias 2024-3 mg	4	51	0	55	2.5%	9.69 [0.53, 175.67]
Frias 2024-45 mg	4	63	0	55	2.7%	7.88 [0.43, 143.07]
Horn 2025-12 mg	38	331	4	628	14.1%	18.02 [6.49, 50.07]
Horn 2025-36 mg	28	321	4	628	13.8%	13.69 [4.85, 38.71]
Horn 2025-6 mg	21	328	4	628	14.0%	10.05 [3.48, 29.04]
Ohwaki 2026-12 mg	2	14	0	11	2.8%	4.00 [0.21, 75.66]
Ohwaki 2026-24 mg	0	17	0	11		Not estimable
Ohwaki 2026-45 mg	1	18	0	11	3.1%	1.89 [0.08, 42.82]
Pratt 2023-15 mg	3	10	0	17	1.9%	11.45 [0.65, 201.35]
Pratt 2023-21 mg	0	14	0	17		Not estimable
Pratt 2023-27 mg	2	9	0	17	1.8%	9.00 [0.48, 169.62]
Pratt 2023-45 mg	2	9	0	17	1.8%	9.00 [0.48, 169.62]
Pratt 2023-9 mg	1	9	0	17	1.8%	5.40 [0.24, 120.57]
Rosenstock 2025-12 mg	10	137	0	138	2.5%	21.15 [1.25, 357.45]
Rosenstock 2025-36 mg	6	141	0	138	2.6%	12.73 [0.72, 223.75]
Rosenstock 2025-3 mg	5	143	0	138	2.6%	10.62 [0.59, 190.23]
Wharton 2023-12 mg	9	50	0	50	2.5%	19.00 [1.14, 317.87]
Wharton 2025-24 mg	11	53	0	50	2.6%	21.72 [1.31, 359.16]
Wharton 2025-36 mg-1	5	29	0	50	1.9%	18.70 [1.07, 326.44]
Wharton 2025-36 mg-2	2	29	0	50	1.9%	8.50 [0.42, 171.19]
Wharton 2025-45 mg-1	2	31	0	50	2.0%	7.97 [0.40, 160.70]
Wharton 2025-45 mg-2	6	30	0	50	1.9%	21.39 [1.25, 366.64]
**Subtotal (95% CI)**		**2001**		**2991**	**88.4%**	**12.55 [8.08, 19.49]**
Total events	175		12			
Heterogeneity: Chi² = 4.40, df = 22 (P = 1.00); I² = 0%						
Test for overall effect: Z = 11.26 (P < 0.00001)						
**Total (95% CI)**		**2203**		**3277**	**100.0%**	**11.62 [7.67, 17.61]**
Total events	182		12			
Heterogeneity: Chi² = 7.00, df = 27 (P = 1.00); I² = 0%						
Test for overall effect: Z = 11.58 (P < 0.00001)						
Test for subgroup differences: Chi² = 2.07, df = 1 (P = 0.15), I² = 51.7%						

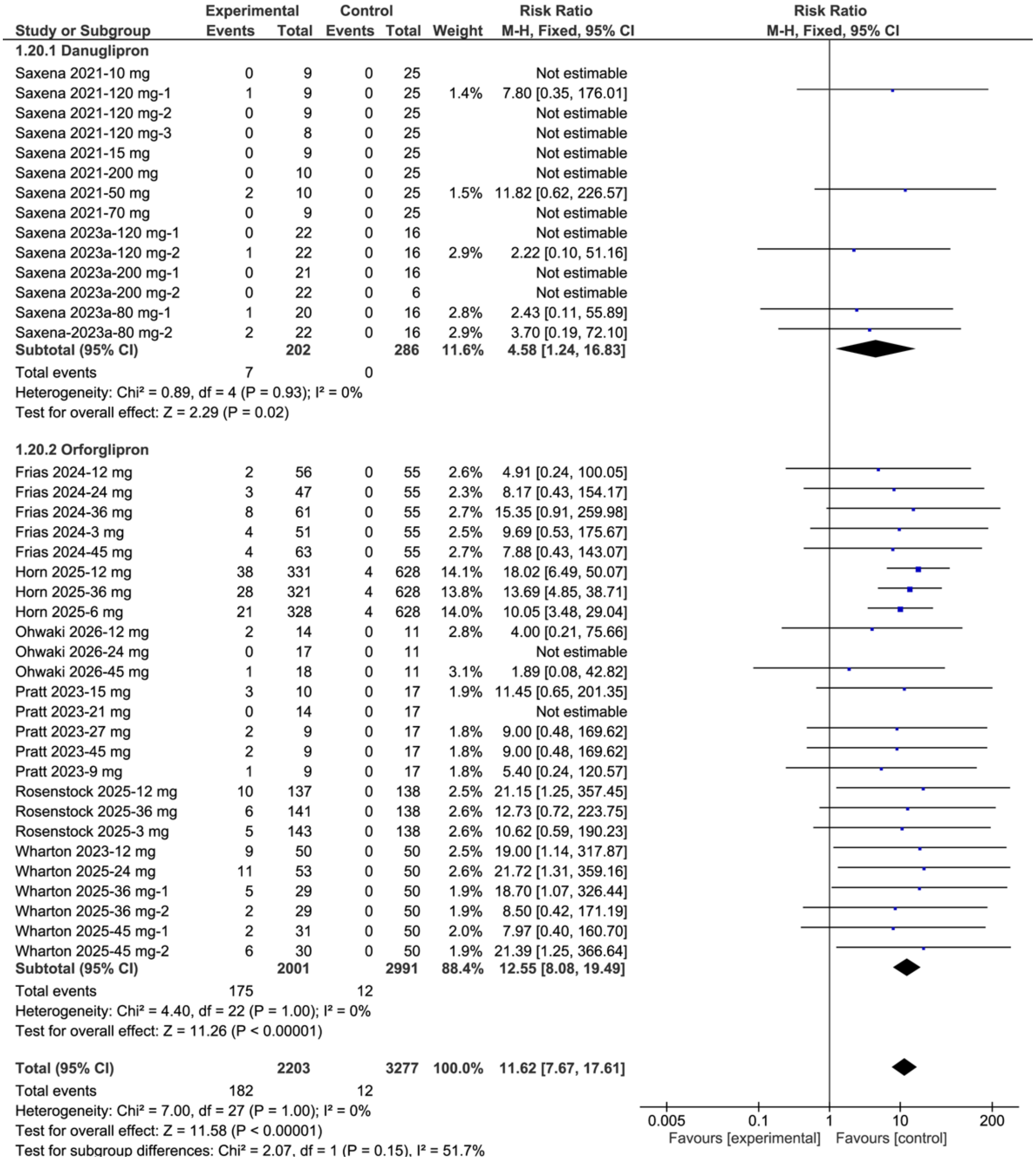

Supplemental Figure S5. Forest plot of eructation. Forest plot showing the pooled RR for eructation associated with oral small-molecule GLP-1 receptor agonists compared with control, with subgroup analyses according to danuglipron and orforglipron.

Study or Subgroup	Experimental Events	Experimental Total	Control Events	Control Total	Weight	Risk Ratio M-H, Fixed, 95% CI
**1.22.1 Danuglipron**						
Saxena 2023a-120 mg-1	1	22	1	16	5.8%	0.73 [0.05, 10.78]
Saxena 2023a-120 mg-2	0	22	1	16	8.6%	0.25 [0.01, 5.68]
Saxena 2023a-200 mg-1	1	21	1	16	5.7%	0.76 [0.05, 11.27]
Saxena 2023a-200 mg-2	1	22	0	6	3.8%	0.91 [0.04, 20.00]
Saxena 2023a-80 mg-1	2	20	1	16	5.5%	1.60 [0.16, 16.10]
Saxena-2023a-80 mg-2	1	22	0	16	2.9%	2.22 [0.10, 51.16]
Saxena 2023b-10 mg	2	68	0	66	2.5%	4.86 [0.24, 99.26]
Saxena 2023b-120 mg	5	71	0	66	2.6%	10.24 [0.58, 181.60]
Saxena 2023b-2.5 mg	1	68	0	66	2.5%	2.91 [0.12, 70.25]
Saxena 2023b-40 mg	2	71	0	66	2.6%	4.65 [0.23, 95.16]
Saxena 2023b-80 mg	4	67	0	66	2.5%	8.87 [0.49, 161.52]
**Subtotal (95% CI)**		**474**		**416**	**44.9%**	**2.44 [1.13, 5.24]**
Total events	20		4			
Heterogeneity: Chi² = 6.16, df = 10 (P = 0.80); I² = 0%						
Test for overall effect: Z = 2.28 (P = 0.02)						
**1.22.2 Orforglipron**						
Rosenstock 2025-12 mg	7	137	2	138	9.9%	3.53 [0.75, 16.67]
Rosenstock 2025-36 mg	5	141	2	138	10.1%	2.45 [0.48, 12.40]
Rosenstock 2025-3 mg	3	143	2	138	10.1%	1.45 [0.25, 8.53]
Wharton 2023-12 mg	4	50	1	50	5.0%	4.00 [0.46, 34.54]
Wharton 2025-24 mg	5	53	1	50	5.1%	4.72 [0.57, 38.98]
Wharton 2025-36 mg-1	3	29	1	50	3.7%	5.17 [0.56, 47.46]
Wharton 2025-36 mg-2	4	29	1	50	3.7%	6.90 [0.81, 58.79]
Wharton 2025-45 mg-1	4	31	1	50	3.8%	6.45 [0.76, 55.11]
Wharton 2025-45 mg-2	2	30	1	50	3.7%	3.33 [0.32, 35.21]
**Subtotal (95% CI)**		**643**		**714**	**55.1%**	**3.62 [1.92, 6.83]**
Total events	37		12			
Heterogeneity: Chi² = 2.05, df = 8 (P = 0.98); I² = 0%						
Test for overall effect: Z = 3.98 (P < 0.0001)						
**Total (95% CI)**		**1117**		**1130**	**100.0%**	**3.09 [1.90, 5.03]**
Total events	57		16			
Heterogeneity: Chi² = 9.14, df = 19 (P = 0.97); I² = 0%						
Test for overall effect: Z = 4.55 (P < 0.00001)						
Test for subgroup differences: Chi² = 0.61, df = 1 (P = 0.44), I² = 0%						

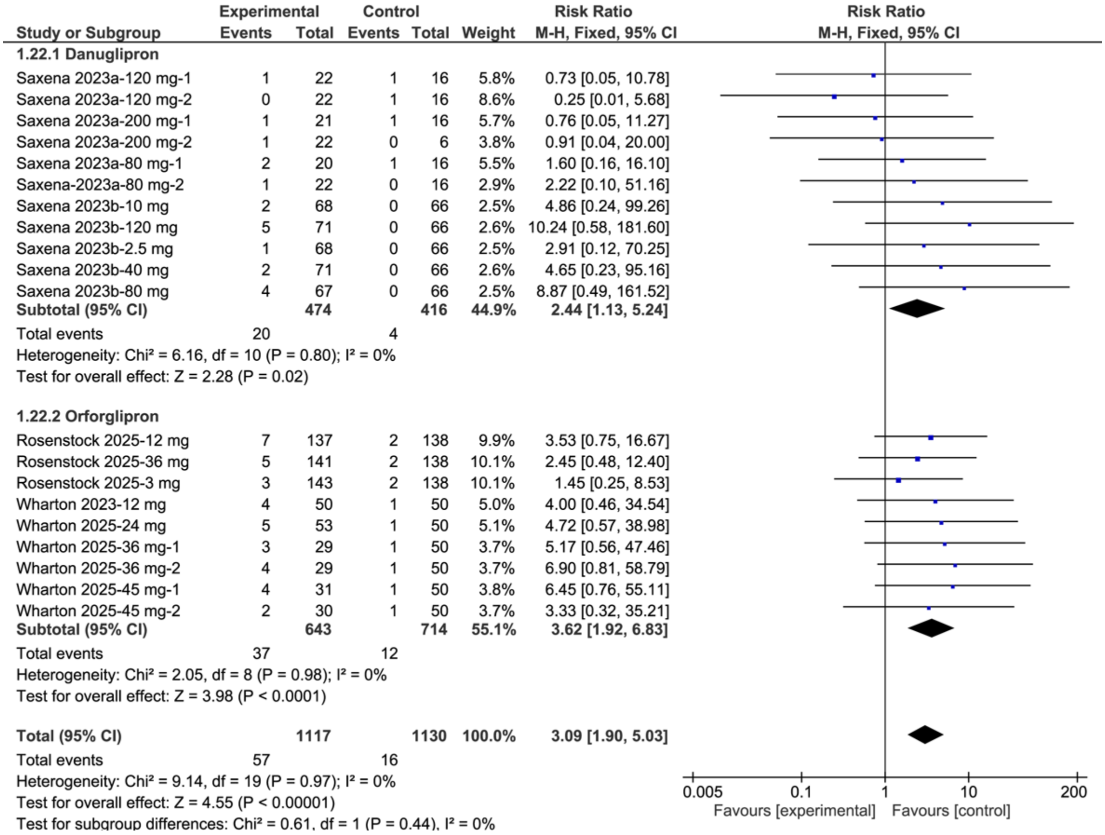

Supplemental Figure S6. Forest plot of gastro-oesophageal reflux disease. Forest plot showing the pooled RR for gastro-oesophageal reflux disease associated with oral small-molecule GLP-1 receptor agonists compared with control, with subgroup analyses according to danuglipron and orforglipron.

Study or Subgroup	Experimental Events	Experimental Total	Control Events	Control Total	Weight	Risk Ratio M-H, Fixed, 95% CI
**1.21.1 Danuglipron**						
Saxena 2021-10 mg	0	9	0	25		Not estimable
Saxena 2021-120 mg-1	0	9	0	25		Not estimable
Saxena 2021-120 mg-2	0	9	0	25		Not estimable
Saxena 2021-120 mg-3	2	8	0	25	0.6%	14.44 [0.76, 273.29]
Saxena 2021-15 mg	0	9	0	25		Not estimable
Saxena 2021-200 mg	0	10	0	25		Not estimable
Saxena 2021-50 mg	0	10	0	25		Not estimable
Saxena 2021-70 mg	3	9	0	25	0.6%	18.20 [1.03, 321.65]
Saxena 2023a-120 mg-1	0	22	0	16		Not estimable
Saxena 2023a-120 mg-2	0	22	0	16		Not estimable
Saxena 2023a-200 mg-1	2	21	0	16	1.3%	3.86 [0.20, 75.28]
Saxena 2023a-200 mg-2	1	22	0	6	1.7%	0.91 [0.04, 20.00]
Saxena 2023a-80 mg-1	1	20	0	16	1.3%	2.43 [0.11, 55.89]
Saxena-2023a-80 mg-2	0	22	0	16		Not estimable
Saxena 2023b-10 mg	1	68	1	66	2.3%	0.97 [0.06, 15.20]
Saxena 2023b-120 mg	2	71	1	66	2.4%	1.86 [0.17, 20.03]
Saxena 2023b-2.5 mg	0	68	1	66	3.5%	0.32 [0.01, 7.81]
Saxena 2023b-40 mg	4	71	1	66	2.4%	3.72 [0.43, 32.42]
Saxena 2023b-80 mg	3	67	1	66	2.3%	2.96 [0.32, 27.69]
**Subtotal (95% CI)**		**547**		**616**	**18.3%**	**2.89 [1.37, 6.08]**
Total events	19		5			
Heterogeneity: Chi² = 5.92, df = 9 (P = 0.75); I² = 0%						
Test for overall effect: Z = 2.80 (P = 0.005)						
**1.21.2 Orforglipron**						
Ohwaki 2026-12 mg	1	14	0	11	1.3%	2.40 [0.11, 53.77]
Ohwaki 2026-24 mg	1	17	0	11	1.4%	2.00 [0.09, 45.12]
Ohwaki 2026-45 mg	0	18	0	11		Not estimable
Pratt 2023-15 mg	1	10	0	17	0.9%	4.91 [0.22, 110.23]
Pratt 2023-21 mg	1	14	0	17	1.0%	3.60 [0.16, 82.05]
Pratt 2023-27 mg	2	9	0	17	0.8%	9.00 [0.48, 169.62]
Pratt 2023-45 mg	2	9	0	17	0.8%	9.00 [0.48, 169.62]
Pratt 2023-9 mg	0	9	0	17		Not estimable
Rosenstock 2025-12 mg	7	137	11	138	24.9%	0.64 [0.26, 1.60]
Rosenstock 2025-36 mg	16	141	11	138	25.3%	1.42 [0.69, 2.96]
Rosenstock 2025-3 mg	6	143	11	138	25.4%	0.53 [0.20, 1.38]
**Subtotal (95% CI)**		**521**		**532**	**81.7%**	**1.15 [0.75, 1.75]**
Total events	37		33			
Heterogeneity: Chi² = 9.84, df = 8 (P = 0.28); I² = 19%						
Test for overall effect: Z = 0.62 (P = 0.53)						
**Total (95% CI)**		**1068**		**1148**	**100.0%**	**1.46 [1.02, 2.10]**
Total events	56		38			
Heterogeneity: Chi² = 19.36, df = 18 (P = 0.37); I² = 7%						
Test for overall effect: Z = 2.06 (P = 0.04)						
Test for subgroup differences: Chi² = 4.48, df = 1 (P = 0.03), I² = 77.7%						

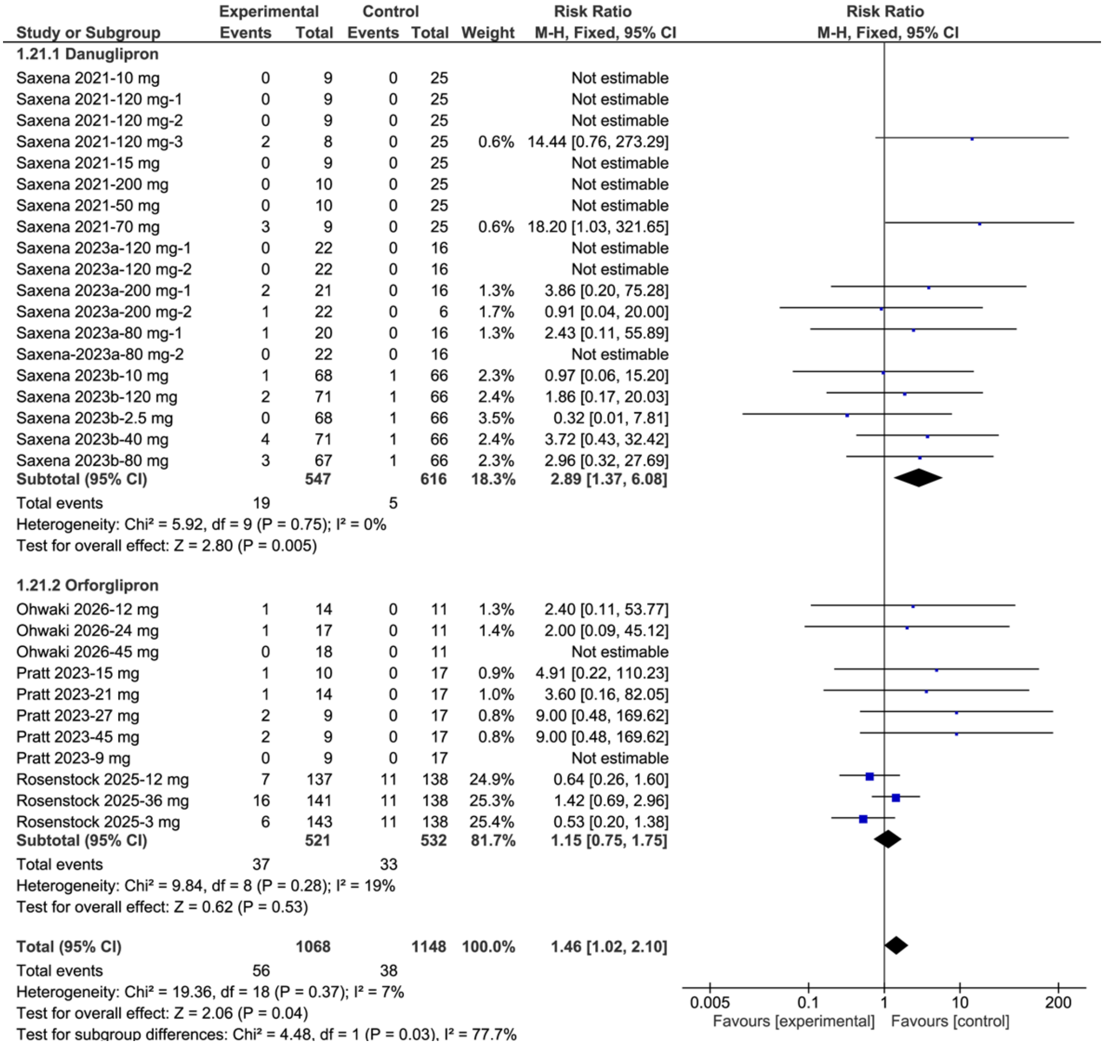

Supplemental Figure S7. Forest plot of abdominal distension. Forest plot showing the pooled RR for abdominal distension associated with oral small-molecule GLP-1 receptor agonists compared with control, with subgroup analyses according to danuglipron and orforglipron.

Study or Subgroup	Experimental Events	Experimental Total	Control Events	Control Total	Weight	Risk Ratio M-H, Fixed, 95% CI
**1.19.1 Danuglipron**						
Saxena 2021-10 mg	0	9	0	25		Not estimable
Saxena 2021-120 mg-1	2	9	0	25	7.9%	13.00 [0.68, 247.75]
Saxena 2021-120 mg-2	2	9	0	25	7.9%	13.00 [0.68, 247.75]
Saxena 2021-120 mg-3	1	8	0	25	7.3%	8.67 [0.39, 194.23]
Saxena 2021-15 mg	0	9	0	25		Not estimable
Saxena 2021-200 mg	0	10	0	25		Not estimable
Saxena 2021-50 mg	0	10	0	25		Not estimable
Saxena 2021-70 mg	0	9	0	25		Not estimable
**Subtotal (95% CI)**		**73**		**200**	**23.1%**	**11.63 [2.06, 65.55]**
Total events	5		0			
Heterogeneity: Chi² = 0.05, df = 2 (P = 0.98); I² = 0%						
Test for overall effect: Z = 2.78 (P = 0.005)						
**1.19.2 Orforglipron**						
Ohwaki 2026-12 mg	1	14	0	11	15.8%	2.40 [0.11, 53.77]
Ohwaki 2026-24 mg	3	17	0	11	17.1%	4.67 [0.26, 82.45]
Ohwaki 2026-45 mg	0	18	0	11		Not estimable
Ono 2023-120 mg	5	9	0	9	14.2%	11.00 [0.70, 173.66]
Ono 2023-40 mg	4	10	0	9	14.9%	8.18 [0.50, 133.66]
Ono 2023-80 mg	4	10	0	9	14.9%	8.18 [0.50, 133.66]
**Subtotal (95% CI)**		**78**		**60**	**76.9%**	**6.73 [1.93, 23.52]**
Total events	17		0			
Heterogeneity: Chi² = 0.64, df = 4 (P = 0.96); I² = 0%						
Test for overall effect: Z = 2.99 (P = 0.003)						
**Total (95% CI)**		**151**		**260**	**100.0%**	**7.87 [2.85, 21.68]**
Total events	22		0			
Heterogeneity: Chi² = 0.97, df = 7 (P = 1.00); I² = 0%						
Test for overall effect: Z = 3.99 (P < 0.0001)						
Test for subgroup differences: Chi² = 0.25, df = 1 (P = 0.62), I² = 0%						

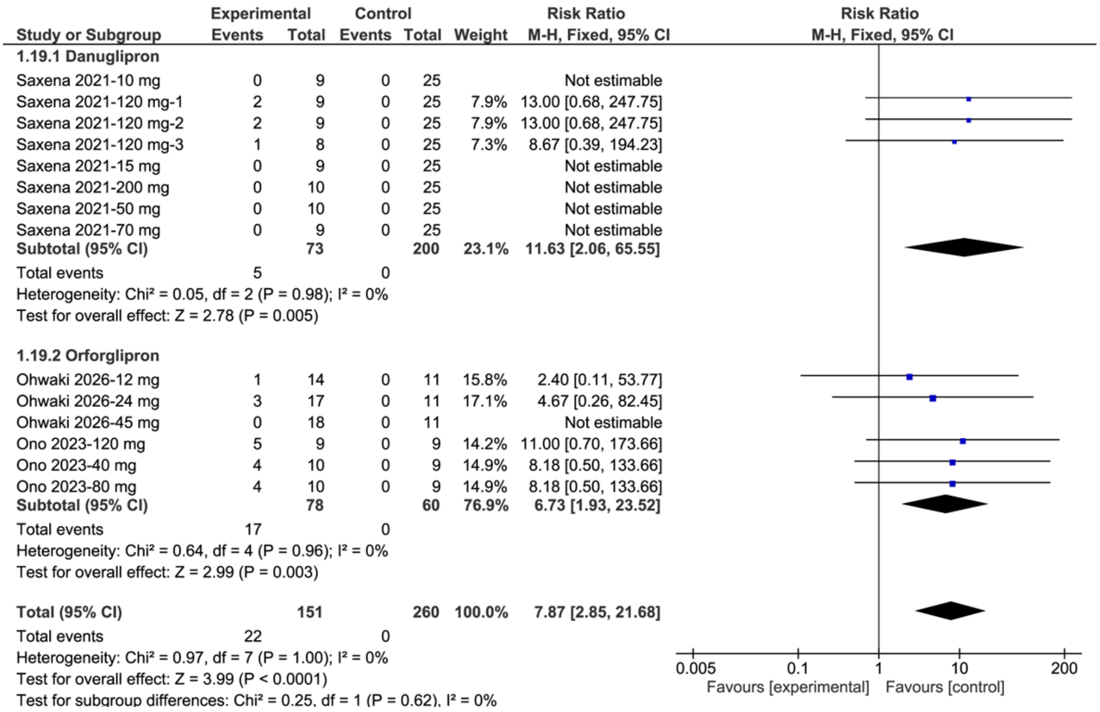

Supplemental Figure S8. Forest plot of abdominal discomfort. Forest plot showing the pooled RR for abdominal discomfort associated with oral small-molecule GLP-1 receptor agonists compared with control, with subgroup analyses according to danuglipron and orforglipron.

Study or Subgroup	Experimental Events	Experimental Total	Control Events	Control Total	Weight	Risk Ratio M-H, Fixed, 95% CI
**1.23.1 Danuglipron**						
Saxena 2023a-120 mg-1	0	22	0	16		Not estimable
Saxena 2023a-120 mg-2	0	22	0	16		Not estimable
Saxena 2023a-200 mg-1	1	21	0	16	1.1%	2.32 [0.10, 53.42]
Saxena 2023a-200 mg-2	0	22	0	6		Not estimable
Saxena 2023a-80 mg-1	2	20	0	16	1.0%	4.05 [0.21, 78.76]
Saxena-2023a-80 mg-2	2	22	0	16	1.1%	3.70 [0.19, 72.10]
**Subtotal (95% CI)**		**129**		**86**	**3.2%**	**3.35 [0.59, 19.04]**
Total events	5		0			
Heterogeneity: Chi² = 0.07, df = 2 (P = 0.96); I² = 0%						
Test for overall effect: Z = 1.36 (P = 0.17)						
**1.23.2 Orforglipron**						
Horn 2025-12 mg	20	331	17	628	22.1%	2.23 [1.19, 4.20]
Horn 2025-36 mg	18	321	17	628	21.7%	2.07 [1.08, 3.96]
Horn 2025-6 mg	19	328	17	628	22.0%	2.14 [1.13, 4.06]
Rosenstock 2025-12 mg	4	137	2	138	3.8%	2.01 [0.38, 10.82]
Rosenstock 2025-36 mg	7	141	2	138	3.8%	3.43 [0.72, 16.20]
Rosenstock 2025-3 mg	1	143	2	138	3.8%	0.48 [0.04, 5.26]
Wharton 2023-12 mg	4	50	2	50	3.8%	2.00 [0.38, 10.43]
Wharton 2025-24 mg	4	53	2	50	3.9%	1.89 [0.36, 9.85]
Wharton 2025-36 mg-1	0	29	2	50	3.5%	0.34 [0.02, 6.85]
Wharton 2025-36 mg-2	2	29	2	50	2.8%	1.72 [0.26, 11.59]
Wharton 2025-45 mg-1	2	31	2	50	2.9%	1.61 [0.24, 10.87]
Wharton 2025-45 mg-2	1	30	2	50	2.8%	0.83 [0.08, 8.80]
**Subtotal (95% CI)**		**1623**		**2598**	**96.8%**	**1.98 [1.44, 2.71]**
Total events	82		69			
Heterogeneity: Chi² = 3.94, df = 11 (P = 0.97); I² = 0%						
Test for overall effect: Z = 4.25 (P < 0.0001)						
**Total (95% CI)**		**1752**		**2684**	**100.0%**	**2.02 [1.48, 2.76]**
Total events	87		69			
Heterogeneity: Chi² = 4.31, df = 14 (P = 0.99); I² = 0%						
Test for overall effect: Z = 4.46 (P < 0.00001)						
Test for subgroup differences: Chi² = 0.34, df = 1 (P = 0.56), I² = 0%						

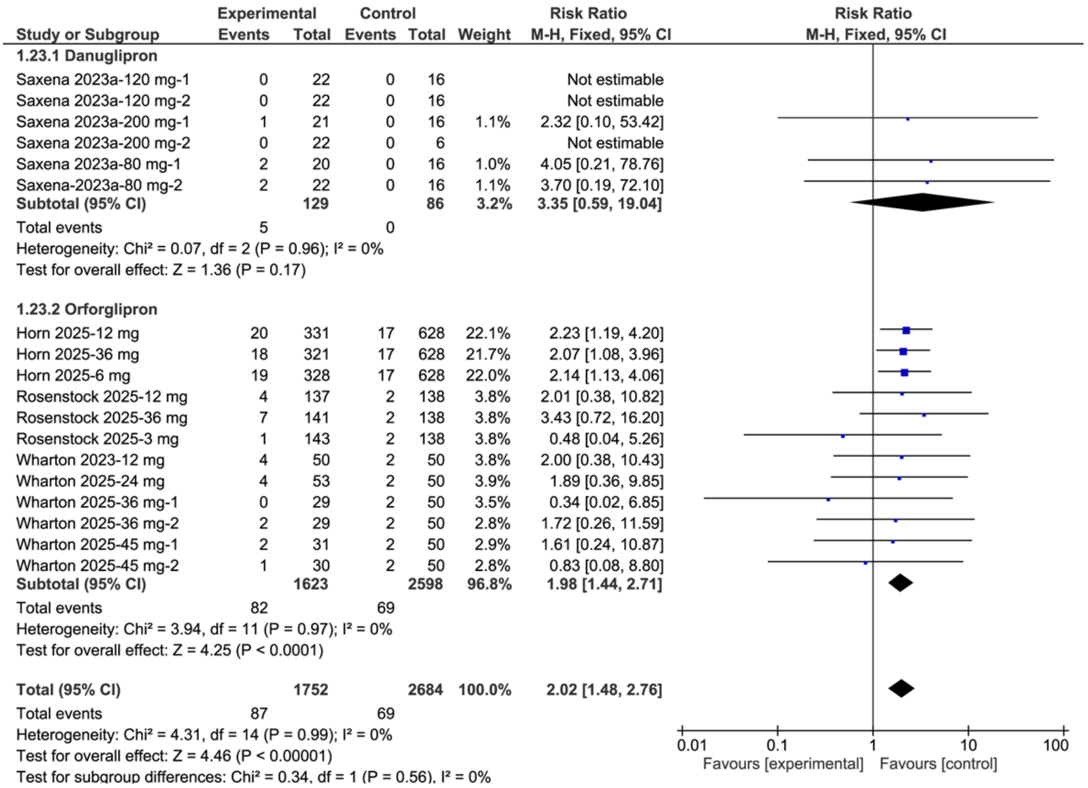

Supplemental Figure S9. Forest plot of abdominal pain. Forest plot showing the pooled RR for abdominal pain associated with oral small-molecule GLP-1 receptor agonists compared with control, with subgroup analyses according to danuglipron and orforglipron.

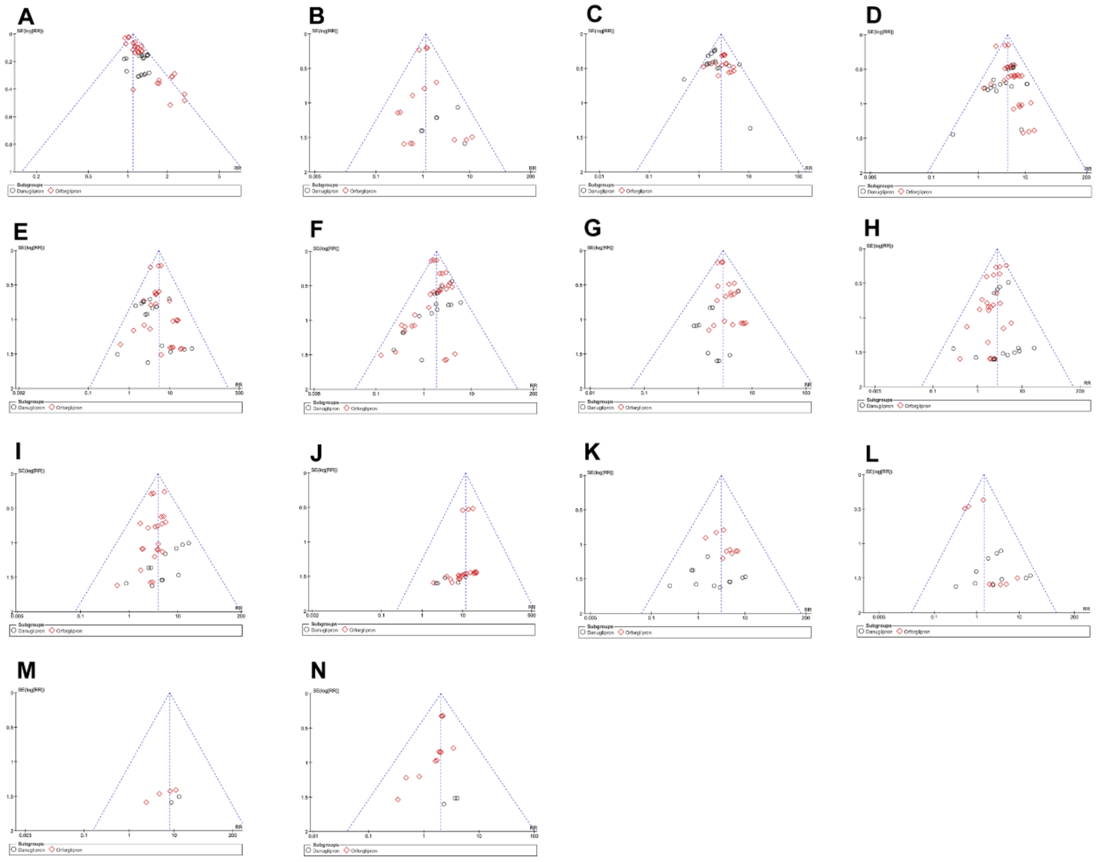

Supplemental Figure S10. Funnel plots for adverse-event outcomes. Funnel plots assessing potential publication bias for the analyzed adverse-event outcomes including (A) TEAEs, (B) AEs, (C) SAEs, (D) Nausea, (E) Vomiting, (F) Diarrhea, (G) Constipation, (H) dyspepsia, (I) decreased appetite, (J) eructation, (K) gastro-oesophageal reflux disease, (L) abdominal distension, (M) abdominal discomfort, (N) abdominal pain.

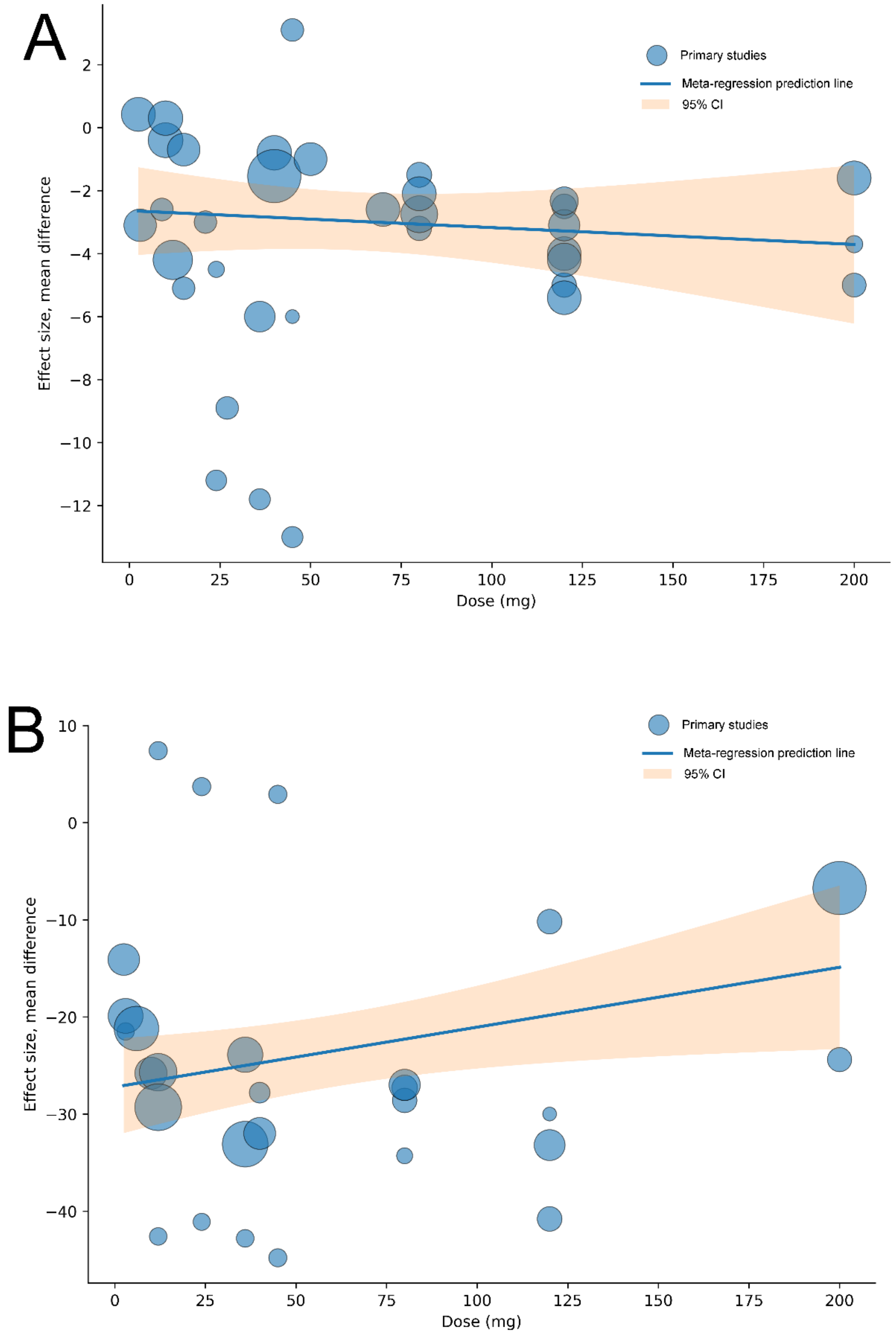

Supplemental Figure S11. Dose–effect meta-regression analyses for body weight change (A) and FBG (B).
